# Structural Differences Explain Diverse Functions of *Plasmodium* Actins

**DOI:** 10.1371/journal.ppat.1004091

**Published:** 2014-04-17

**Authors:** Juha Vahokoski, Saligram Prabhakar Bhargav, Ambroise Desfosses, Maria Andreadaki, Esa-Pekka Kumpula, Silvia Muñico Martinez, Alexander Ignatev, Simone Lepper, Friedrich Frischknecht, Inga Sidén-Kiamos, Carsten Sachse, Inari Kursula

**Affiliations:** 1 Faculty of Biochemistry and Molecular Medicine, University of Oulu, Oulu, Finland; 2 European Molecular Biology Laboratory, Structural and Computational Biology Unit, Heidelberg, Germany; 3 Institute of Molecular Biology and Biotechnology, Foundation for Research and Technology – Hellas, Heraklion, Crete, Greece; 4 Centre for Structural Systems Biology; Helmholtz Centre for Infection Research and German Electron Synchrotron, Hamburg, Germany; 5 Parasitology – Department of Infectious Diseases, University of Heidelberg Medical School, Heidelberg, Germany; MRC National Institute for Medical Research, United Kingdom

## Abstract

Actins are highly conserved proteins and key players in central processes in all eukaryotic cells. The two actins of the malaria parasite are among the most divergent eukaryotic actins and also differ from each other more than isoforms in any other species. Microfilaments have not been directly observed in *Plasmodium* and are presumed to be short and highly dynamic. We show that actin I cannot complement actin II in male gametogenesis, suggesting critical structural differences. Cryo-EM reveals that *Plasmodium* actin I has a unique filament structure, whereas actin II filaments resemble canonical F-actin. Both *Plasmodium* actins hydrolyze ATP more efficiently than α-actin, and unlike any other actin, both parasite actins rapidly form short oligomers induced by ADP. Crystal structures of both isoforms pinpoint several structural changes in the monomers causing the unique polymerization properties. Inserting the canonical D-loop to *Plasmodium* actin I leads to the formation of long filaments *in vitro*. *In vivo*, this chimera restores gametogenesis in parasites lacking actin II, suggesting that stable filaments are required for exflagellation. Together, these data underline the divergence of eukaryotic actins and demonstrate how structural differences in the monomers translate into filaments with different properties, implying that even eukaryotic actins have faced different evolutionary pressures and followed different paths for developing their polymerization properties.

## Author Summary

Malaria parasites have two actin isoforms, which are among the most divergent within the actin family that comprises highly conserved proteins, essential in all eukaryotic cells. In *Plasmodium*, actin is indispensable for motility and, thus, the infectivity of the deadly parasite. Yet, actin filaments have not been observed *in vivo* in these pathogens. Here, we show that the two *Plasmodium* actins differ from each other in both monomeric and filamentous form and that actin I cannot replace actin II during male gametogenesis. Whereas the major isoform actin I cannot form stable filaments alone, the mosquito-stage-specific actin II readily forms long filaments that have dimensions similar to canonical actins. A chimeric actin I mutant that forms long filaments *in vitro* also rescues gametogenesis in parasites lacking actin II. Both *Plasmodium* actins rapidly hydrolyze ATP and form short oligomers in the presence of ADP, which is a fundamental difference to all other actins characterized to date. Structural and functional differences in the two *Plasmodium* actin isoforms compared both to each other and to canonical actins reveal how the polymerization properties of eukaryotic actins have evolved along different avenues.

## Introduction

Actins are the most abundant and among the most conserved proteins in eukaryotic cells and play indispensable roles in a plethora of key cellular events, including muscle contraction, cell division, shape determination, transport, and cell motility [1], [2]. Actins are highly conserved in opisthokonts with <10% divergence between yeast and man. The six mammalian actin isoforms differ from each other by a maximum of 6% of the sequence, and are virtually identical across species. Nevertheless, these subtle differences are enough to determine isoform-specific functions [3]. Common to most actins is their capacity to form long filaments. However, in a number of phylogenetically distinct organisms, such as *Trypanosoma* and *Plasmodium spp.*, actin filaments have not been observed [4], [5]. Unlike other members of the phylum *Apicomplexa*, which comprises single-celled eukaryotic intracellular parasites, the malaria parasites have two actin isoforms, which at the sequence level are <80% identical with canonical (opisthokont) actins and each other. This is a remarkable difference, considering the near identity among canonical actins (**Fig. S1**). An important question is how this divergence at the amino-acid level translates into different structures – and how this, in turn, influences polymerization.

Most studies on apicomplexan actins have concentrated on their role in gliding motility, a unique mode of migration, essential for the parasite to infect new cells. However, like in other eukaryotes, parasite actins must have several cellular functions. Actin polymerization is indispensable for gliding and likely involved in host cell invasion and egress [6]–[8]. Despite evidence for this crucial role of filamentous actin, long filaments have only been visualized in *Theileria* [9], which appears not to use actin filaments for host cell invasion [10]. The presence of regular actin filaments in *Plasmodium* is uncertain [11]–[13]. *In vitro*, apicomplexan actins form short, ∼100-nm long filaments, which undergo rapid treadmilling [14]–[17]. Recently, specific antibodies revealed filament-like structures in motile forms of *Plasmodium* [12], [13]. *Toxoplasma gondii* actin, which is 93% identical to *Plasmodium* actin I, has been reported to polymerize at concentrations 10-fold lower than canonical actins [15], and most recently, it has been proposed to polymerize in an isodesmic manner without a lag phase or a critical concentration, which is unique among all actins or actin homologs studied to date [18]. Yet, most of the cellular actin is present as monomers [19], implying that filaments occur only transiently, and polymerization is under tight control of regulatory proteins or governed by distinct properties of the monomer. On the other hand, it has been estimated that 2/3 of *Plasmodium* actin in merozoites – the infective blood stage form, which does not exhibit gliding motility – could be present as short filaments [20].

*Plasmodium* actin I is abundant and expressed throughout the life cycle of the parasite, whereas actin II is present only in the gametocytes and mosquito stages [21]–[24], including sporozoites [25], the highly motile form of the parasite, transmitted to the vertebrate by the mosquito. Actin I is an indispensable part of the parasite motor machinery responsible for the unique gliding motility of the parasite. Actin II has at least two functions in the mosquito stages, as revealed by reverse genetics analyses. It is required both during male gametogenesis and in the zygote stage [23], [24]. However, no clear molecular function has been assigned for actin II.

To understand the properties of the divergent *Plasmodium* actins, we have determined their monomer crystal structures and analyzed their filament assembly using electron microscopy (EM). We show that, unlike in any other cell reported so far, the two isoforms differ substantially from each other in their ability to form filaments and that both oligomerize in the presence of ADP. Their functional uniqueness is further highlighted by the finding that *Plasmodium* actin II has a distinct role in male gametogenesis that cannot be complemented by actin I. Finally, we show that a chimera of *Plasmodium* actin I and canonical actin can form long filaments and, importantly, restores the function of actin II in gametogenesis.

## Results

### The two *Plasmodium* actins have different filament structures

**Figure 1:**
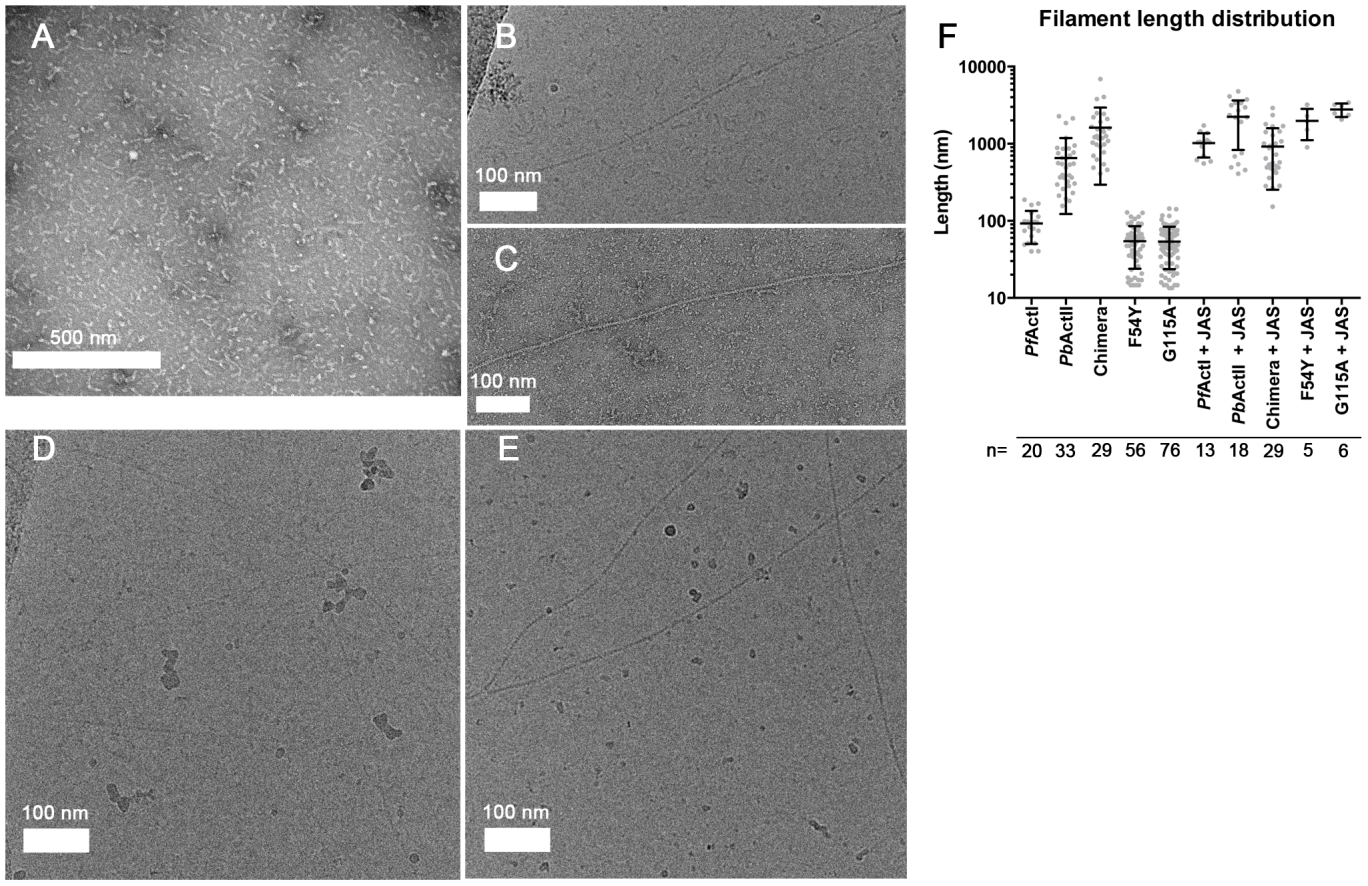
Electron micrographs of *Plasmodium* actin filaments. (**A**) In the absence of stabilizing agents, actin I forms only short structures lacking helical symmetry. (**B,C**) Actin II readily forms filaments varying from hundreds of nm to 1–2 µm in length. (**D,E**) In the presence of JAS, both parasite actins form long helical filaments. (**F**) Length distributions of two *Plasmodium* actin isoforms and three actin I mutants. Note the logarithmic scale of the Y axis.

The most peculiar property of apicomplexan actins is their apparent inability to form long, stable filaments. This is a fundamental difference to all actins studied so far, and in the lack of structural information, the reasons for the poor polymerizability are not understood. It has been shown by atomic force microscopy that the dimensions of jasplakinolide (JAS)-stabilized *P. falciparum* actin I filaments, purified from merozoites, are different with respect to their helical symmetry from canonical actins [26]. However, the structure of actin II filaments has not been studied before. We visualized the structures formed by both *Plasmodium* actin isoforms using EM (**Fig. 1**). When polymerized in the presence of ATP in high-salt conditions at room temperature overnight, actin I forms only short, irregular structures of approximately 100 nm in length (**Fig. 1 A and F**), while actin II forms significantly longer filaments (average length 650 nm) (**Fig. 1 B,C,F**). In the presence of JAS, which *in vitro* stabilizes actin filaments, both actin I and II form long, rather straight filaments (**Fig. 1 D–F**).

**Figure 2:**
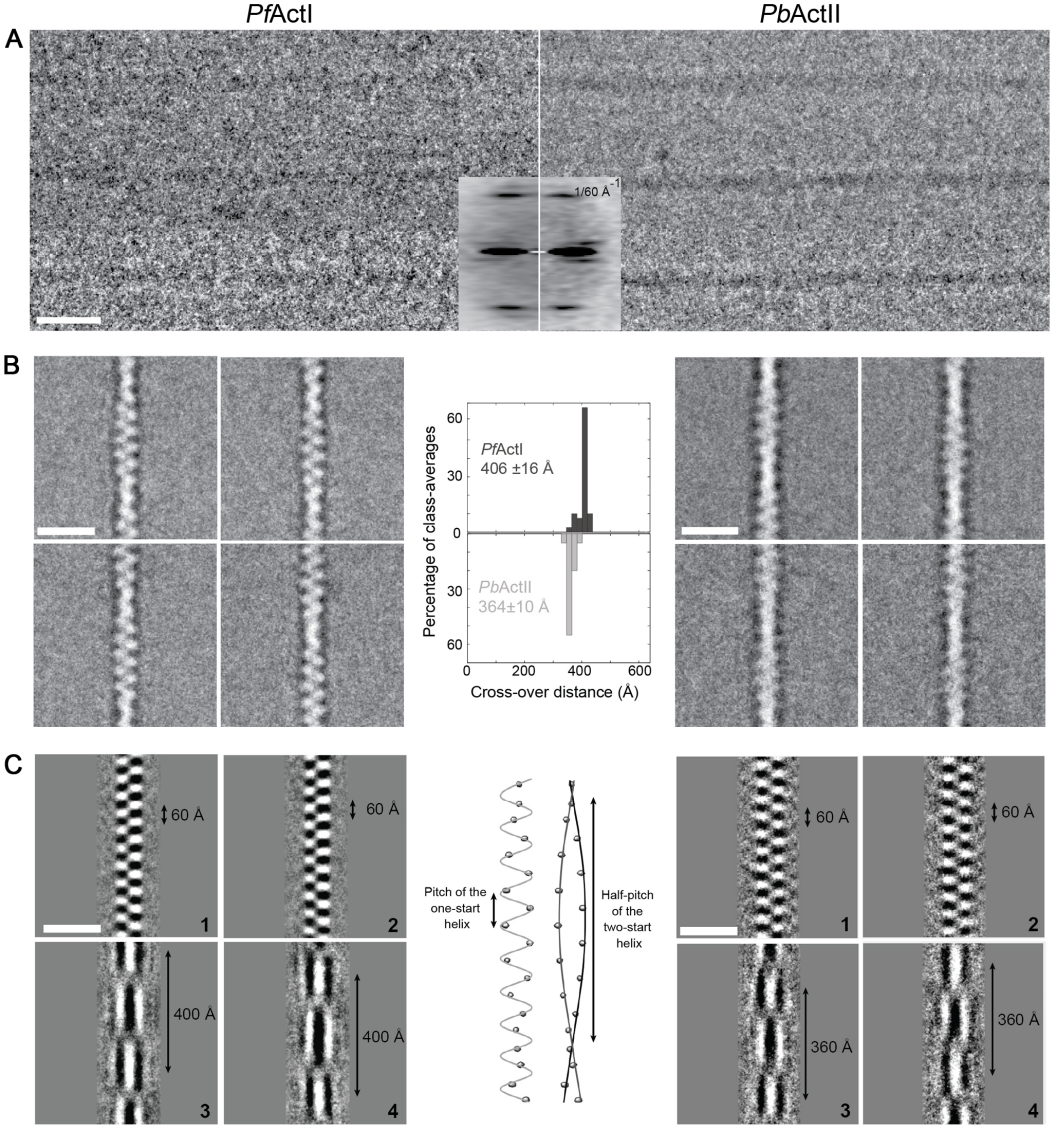
Cryo-EM image analysis of actin I and II. (**A**) Electron cryo-micrographs of actin I and II (left and right, respectively). Side-by-side average power spectrum of actin I and II. (**B**) Representative class averages from k-means clustering. Center: Histograms of measured cross-over distances reveal a larger half pitch for actin I. (**C**) Eigen images 1–2 from actin I and II k-means clustering reveal a constant pitch of the one-start helix, whereas Eigen images 3–4 confirm the difference in cross-over distance of actin I and II.

To evaluate whether the helical assemblies of the two *Plasmodium* actins are different, we used cryo-EM. Filaments of both parasite actins were embedded in vitreous ice (**Fig. 2**). First, we inspected the averaged power spectra derived from segments of 330 and 56 filaments for actin I and II, respectively. These look virtually identical because they can only be compared at 1/60 Å^−1^ resolution (**Fig. 2 A**). We then characterized the structure of the filaments in real space and performed k-means classification of helical segments [27]. Inspection of the classes allows a direct measurement of the cross overs or half-pitch of the two-start helix, which represent the distance the filament requires to undergo a 180° rotation. For actin I and II, cross-over distances cluster around 406±16 Å and 364±10 Å, respectively (**Fig. 2 B**), which is confirmed from Eigen images that represent the half-pitch of the two-start helix (**Fig. 2 C**). Hence, actin II has symmetry parameters identical to α-actin, for which a cross-over distance of 371 Å has been reported [28], while actin I possesses a significantly longer cross-over distance, which is in agreement with the earlier work performed on actin I using atomic force microscopy [26].

**Figure 3:**
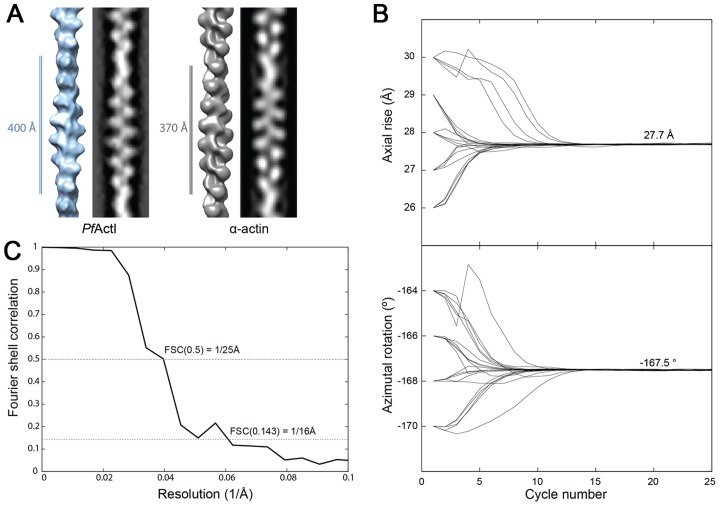
Filament structure of *Plasmodium* actin I compared to α-actin. (**A**) The cryo-EM structure of actin I filament at 25 Å resolution (left) in comparison with rabbit skeletal muscle α-actin filtered to a comparable resolution (right; EM database entry EMD-5168 [28]). (**B**) Symmetry refinement of actin I confirms that the change in cross-over distance is caused mainly by a change in helical rotation when compared with actin II and canonical rabbit α-actin. (**C**) Fourier Shell correlation of actin I half data sets used for 3D reconstruction. The resolution can be estimated at 25 Å based on the 0.5 criterion.

To understand whether the longer cross-over distance is a result of a change in the helical rise and/or the helical rotation, we determined the low-resolution 3D structure of actin I (**Fig. 3 A**) using single-particle based helical reconstruction [29], [30]. We refined the helical symmetry (**Fig. 3 B**) and determined the low-resolution filament structure at 25-Å resolution (at FSC 0.5 cutoff, **Fig. 3 C**). This showed that the cross-over distance change is mainly due to a change of helical rotation from −166.6° [28] to −167.5°, which corresponds to the predicted rotation change if the helical rise remains constant at 27.7 Å. Despite the obvious difference in helical symmetry, at the current resolution, the cryo-EM structure of the actin I subunit looks very similar to the canonical actin filament (**Fig. 3 A**). This is the first time that such large differences in the properties of filaments have been observed for actin isoforms of any species. Thus, higher resolution is needed to further characterize the molecular interactions giving rise to the different polymerization propensities and the observed helical rotation changes.

### Interface regions are the most divergent in *Plasmodium* actin monomers

The divergent polymerization properties of *Plasmodium* actins *in vivo* may be partly accounted for by differences in the activities of the actin-binding proteins, which in these parasites are also poorly conserved and have partially divergent functions compared to canonical counterparts [31]–[38]. However, differences in the actin monomer structure must be responsible for the observed differences in filament structure *in vitro* and also for interactions with regulatory proteins and, thus, functional differences *in vivo*. Therefore, high-resolution structures are required for understanding the biological and molecular differences of the parasite actins compared to their canonical homologs. Such information will also aid us in evaluating the suitability of *Plasmodium* actins as drug targets.

**Figure 4:**
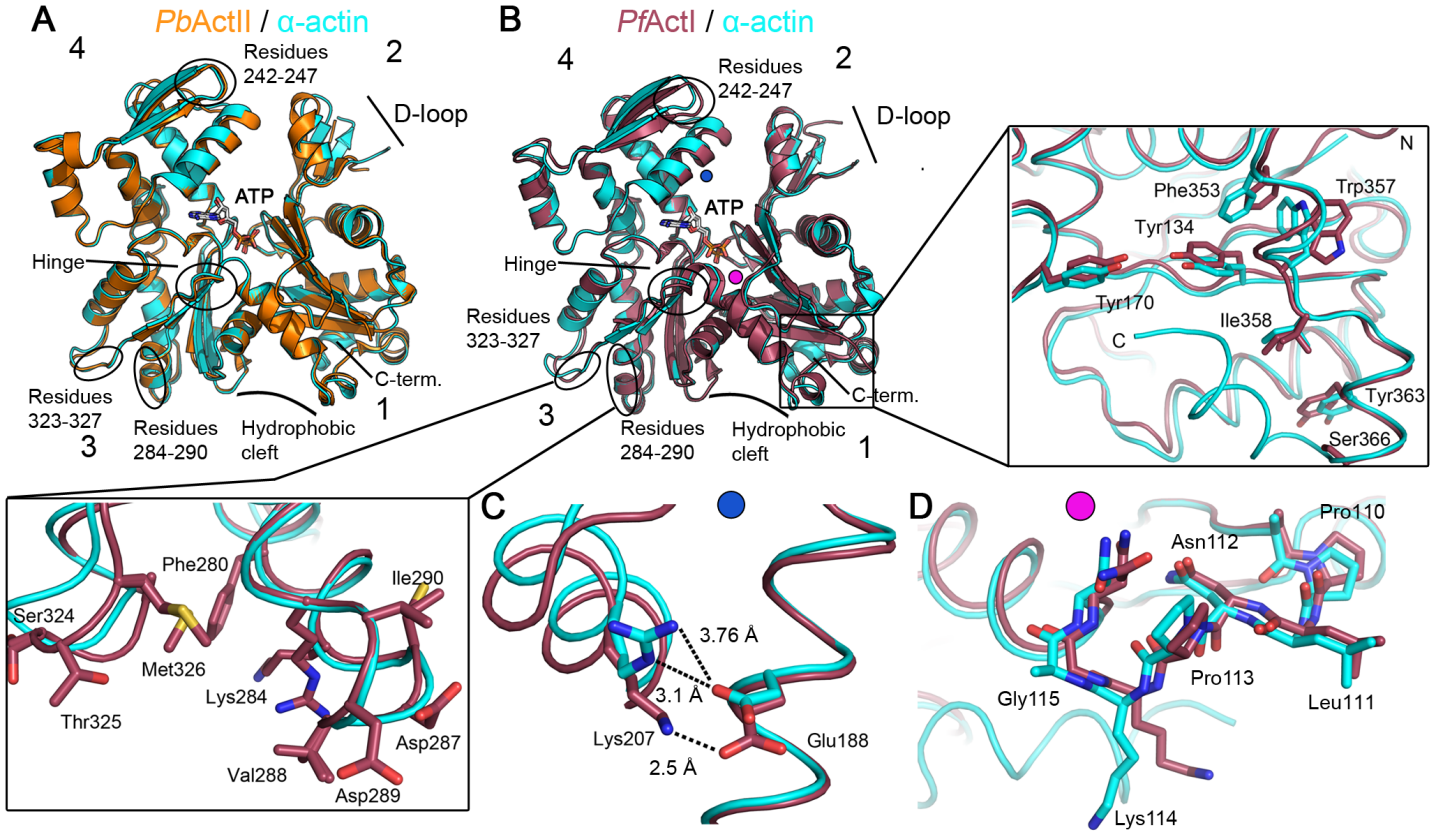
Crystal structures of *Plasmodium* actin I and II. (**A**) *P. berghei* actin II (*Pb*ActII; yellow) superimposed on α-actin (1eqy [39]; cyan). (**B**) *P. falciparum* actin I (*Pf*ActI; red) superimposed on α-actin. In both (**A**) and (**B**), ATP, subdomains 1–4, and several regions discussed in the text are indicated. Both N and C termini reside in subdomain 1; the N terminus is visible at the front, and the C-terminal helix is on the back side. Note that the C-terminal helix is not visible in actin I. The C-terminal part and the nearby hydrophobic cluster with Trp357 are shown in the zoomed view on the right and the region involved in intra-filament contacts in subdomain 3 in the box at the lower left corner. The blue and pink dots in (**B**) indicate the approximate positions of the structural elements shown in detail in (**C**) and (**D**), respectively. (**C**) Lys 207 and Glu188 are at an intimate distance in actin I. A similar salt bridge is present between the corresponding residues in latrunculin-bound α-actin, but the hydrogen-bonding distance is longer without the drug. (**D**) The proline-rich loop with Gly115 in actin I superimposed on that of α-actin. Note the bending of the loop in actin I, due to the more flexible glycine residue.

We set out to determine the crystal structures of *P. falciparum* actin I and *P. berghei* actin II. The sequence identities between the counterparts from *P. falciparum* and *P. berghei* are 99% for actin I and 92% for actin II. The gelsolin G1 domain was used to stabilize the monomers and facilitate crystallization of both actins [39], [40]. The actin I structure was refined to a resolution of 1.3 Å and actin II to 2.2 Å. These high-resolution structures allow for a very detailed comparison of the *Plasmodium* actins with each other and with other actins (**Figs. 4** **, S2, and S3**). Although *Plasmodium* lacks a gelsolin homolog, the mammalian G1 is bound between subdomains 1 and 3 in both *Plasmodium* actins, similarly to other actin–G1 complexes [39], [41] (**Fig. S2 D and E**). For all comparisons, we have used canonical actin structures that have also been determined in complex with G1, in order to rule out structural rearrangements caused by gelsolin binding.

In most canonical actin structures, the C terminus is folded as an α-helix, which interacts with the bottom part of subdomain 1. This is true also for *Plasmodium* actin II (**Fig. 4 A**). In actin I, however, the C terminus turns towards solvent and is disordered (**Fig. 4 B**). The large hydrophobic cleft between subdomains 1 and 3 is defined as a ‘hotspot’ for regulatory protein binding [42] (**Fig. 4 A and B**). A smaller hydrophobic patch in the direct vicinity of the C terminus is, in addition, involved in binding at least profilin [43]. In actin I, the large hydrophobic residues in this smaller cleft, including Trp357, have adopted different conformations compared to canonical actins (**Fig. 4 B**), possibly influencing the binding of profilin, which we have previously proposed to bind actin in a different manner in *Plasmodium* compared to opisthokonts [31]. In actin II, these hydrophobic residues, like the C terminus, are in the canonical conformations. All in all, despite the obvious similarity to other actins, especially actin I shows appreciable structural deviations, in particular in regions involved in binding of regulatory proteins [42]. This may provide possibilities for structure-based drug design targeted at the *Plasmodium* actin–regulatory protein interfaces.

### Residues involved in longitudinal contacts in F-actin differ equally from canonical actins in both parasite actins

A key question concerning the properties of the major *Plasmodium* actin isoform, as well as other apicomplexan actins, is why they, unlike all the extensively studied actins, form only short, unstable filaments. Comparing the crystal structures of the monomers to the recently determined high-resolution cryo-EM structures of canonical actin filaments [28], [44]–[46] can provide clues to answer this question. In canonical F-actin, the axial interactions, meaning the longitudinal contacts between the actin monomers in each of the two protofilaments, are tight and mainly electrostatic, as revealed by cryo-EM studies [28], [45]. The most important axial interactions are discussed below.

The DNase I binding (D-)loop in subdomain 2 (residues 39–61 in actin I) is one of the most important regions for polymerization and, in the filament, inserts into the hydrophobic cleft between subdomains 1 and 3 of the neighboring monomer [28], [45], [47], [48]. In both *Plasmodium* actin crystal structures, the D-loop is disordered, and the tip of it is not visible in the electron density maps (**Figs. 4** **and S3**). Interestingly, the most notable changes in the D-loop sequence concern the first and the last residues of a segment (residues 42–49) that forms a short α-helix in some G-actin structures with ADP bound [49] and has been modeled as a helix also in one of the recent F-actin structures [45]. This part of the D-loop inserts deep into the neighboring monomer in the filament, contacting the so-called proline-rich loop (residues 109–115). Residue 42, which is a glutamine or threonine in most other actins, is a proline in both *Plasmodium* actins. A proline restricts the conformation of the chain and, although also known as a ‘helix breaker’, is often also seen as the first residue in α-helices. At the C terminus of this segment, residue 49, which is a glycine in canonical actins, is a glutamate in both *Plasmodium* actins. These replacements at both ends of this segment would be expected to increase the helical propensity of the tip of the D-loop.

In canonical F-actin, Thr325 in the loop between α-helix 11 and β-strand 18 (residues 323–327; **Fig. 4 A and B**) in subdomain 3 of one monomer interacts with Glu242 in the loop connecting β-strands 15 and 16 in subdomain 4 (residues 242–247; **Fig. 4 A and B**) of the neighboring monomer [28]. In actin I, this loop and the threonine side chain have turned away from the optimal position for this interaction (**Figs. 4 B** **and S3**). In actin II, Ser325 is positioned such that hydrogen bonding to the apposing glutamate can be easily achieved. The difference in the conformation can be explained by the substitutions Y280F and M284K in actin I compared to canonical actins and actin II.

The third main site contributing to axial interactions is formed by the loop connecting helices 9 and 10 (residues 284–290 in subdomain 3; **Fig. 4 A**) inserting between subdomains 2 and 4 of the neighboring monomer. This area is almost fully conserved in both parasite actins, the only substitution being that of Met283 by a lysine (284) in actin I.

All in all, the largest differences in the axial contacts concentrate to the D-loop and concern equally both *Plasmodium* actins. Smaller differences in subdomain 3 may, however, partly explain the different polymerization propensities of the two parasite actin isoforms.

### Filament lateral contact areas display the largest differences between the parasite actins

The lateral contacts between the two protofilaments concern mainly interactions between subdomains 1 and 4 and the beginning of the D-loop in subdomain 2 interacting with the so-called hydrophobic loop (residues 263–275) (**Figs. 4 A–B** **, S1, and S2 D–E**) between subdomains 3 and 4 of the apposing monomer [28], [45], [48]. In a recent high-resolution cryo-EM filament structure [45], Arg206 in subdomain 4 interacts with Ser271 of a neighboring monomer. In actin I, Ser271 is replaced by Ala272, and Lys207 and Glu188 are involved in a short hydrogen bond with salt-bridge character (**Fig. 4 C**). A similar interaction occurs between Arg206 and Asp187 in Latrunculin A-bound α-actin [50]. In the absence of latrunculin, the distance between these residues in α-actin is longer and the geometry suboptimal for a salt bridge (**Fig. 4 C**). Latrunculins prevent actin polymerization, presumably by limiting the flexibility of subdomains 2 and 4 [50],[51], and the salt bridge seen in the latrunculin-actin structure, similar to *Plasmodium* actin I, may be one reason for this. The longer side chain of Glu188 in actin I compared to Asp187 in canonical actins may facilitate the interaction with Lys207. Actin II has Tyr187 in place of Asp187/Glu188, which affects the orientation of Arg206. Actin II, furthermore, has Cys272 in the place of Ser271 of canonical actins, allowing for hydrogen bonding upon polymerization.

At the N terminus of the D-loop, Arg40 and His41 of canonical actins are replaced by lysine and asparagine in actin I and lysine and methionine in actin II. In the filament, Arg40 forms a salt bridge with Glu271 [28], and the replacement to lysine can subtly weaken this interaction. His41, in turn, interacts with Ser266 [28]. Although the asparagine in actin I can also contribute to a hydrogen bond, the interaction may be weaker due to the shorter side chain. In F-actin, Lys114 in the proline-rich loop (residues 109–115 in subdomain 1, following actin I numbering) forms a salt bridge with Glu196 of a neighboring monomer [28]. Uniquely, the neighboring residue is Gly115 in actin I (**Fig. 4 D**), which can have a large effect on the mobility of the proline-rich loop. Actin II has a non-glycine residue (threonine) at this position, similarly to canonical actins (alanine or serine).

Taken together, especially residues involved in lateral contacts, crucial for the stability of the filaments, are altered in *Plasmodium* actin I but more conserved in actin II. The most important differences concern subdomain 4, the D-loop, and the proline-rich loop. The conformations of all these affect each other allosterically and can also modulate the ATPase activity [52]–[54].

### α-actin D-loop rescues polymerization of *Plasmodium* actin I

**Figure 5:**
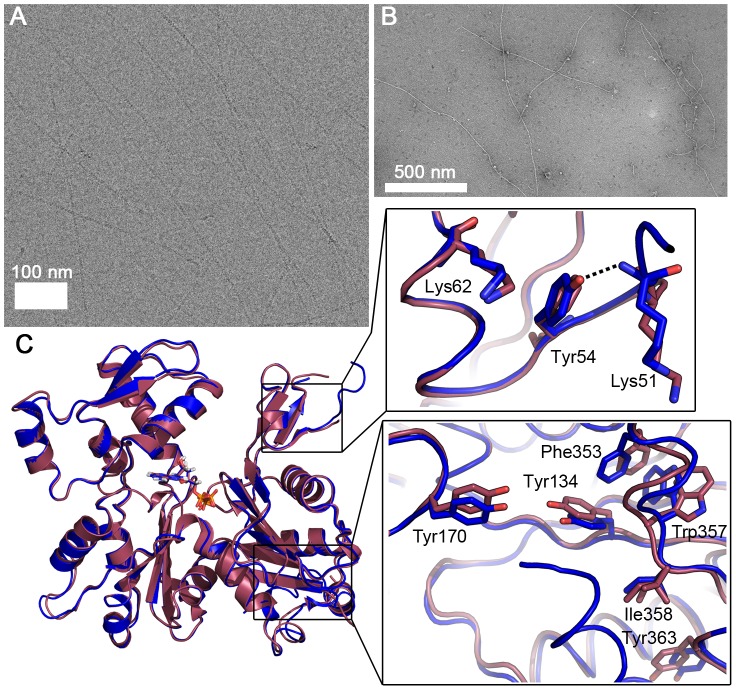
*Plasmodium* actin I–α-actin chimera forms long filaments. (**A**) Electron cryo-micrograph of the chimera filaments. (**B**) Negatively stained chimera filaments. (**C**) The crystal structure of the chimera (blue) resembles that of wild-type actin I (red). The zoomed views show the differences in the D-loop around Tyr54 (above) and the C-terminal helix and the hydrophobic residues nearby, which are in the canonical orientation in the chimera, unlike in actin I (below).

Because of the observed differences in sequence, conformation, and helical symmetry between *Plasmodium* and canonical actins and the importance of the D-loop-mediated contacts for polymerization, we constructed a chimera, in which the entire D-loop of actin I was replaced with that of α-actin. Strikingly, this chimera in the absence of any stabilizing agents forms filaments with an average length of 1.6 µm, which is longer than either actin I or II filaments (**Figs. 5 A–B** **and** **1 F**). However, the appearance of the chimera filaments is not as regular as the filaments formed by canonical actins or actin II.

To further characterize the filaments formed by the actin I–α-actin chimera, we analyzed electron cryo-micrographs of JAS-stabilized chimera filaments and subjected them to the same classification and symmetry analysis as outlined above (**Fig. S4**). Based on the determined half-pitch distance and the symmetry analysis, we conclude that the symmetry parameters of the chimera filaments are in close agreement with the wild-type actin I filaments. Therefore, the symmetry parameters appear to be retained from the wild-type actin I, and thus, the D-loop of canonical actin merely confers increased filament stability.

The crystal structure reveals that the 3D structure of the chimera in monomeric form is very similar to that of wild-type actin I (**Figs. 5 C** **and S3**). However, there are some notable differences. The D-loop in the chimera is slightly more ordered. In addition, the C-terminal helix is visible and can be superimposed with the C terminus of actin II and most canonical actins. The Trp357 side chain in the hydrophobic patch is not flipped as in wild-type actin I (**Figs. 4 B** **and** **5 C**). This observation is in line with earlier reports on allostery between the conformations of the D-loop and the C terminus [55], [56] and demonstrates the ability of even large residues in the hydrophobic core of actin to move, which is a requirement for the rearrangements taking place in the monomer upon polymerization.

In addition to residues already discussed, a notable difference between the D-loop of *Plasmodium* actin I and canonical actins is at position 54, which has a tyrosine in opisthokont actins and *Plasmodium* actin II but phenylalanine in actin I. In the chimera, the OH group of Tyr54 is hydrogen bonded to the main chain of Lys51 and could also interact with the tip of Lys62 (**Fig. 5 C**). In wild-type actin I, Phe54 has moved away from Lys62 and seems to push Lys51 to a slightly different conformation. In actin II, Tyr53 (corresponding to Phe54 in actin I) is able to interact with Lys61 (Lys62 in actin I), but otherwise, this region is rather different in conformation compared to both actin I and canonical actins. This is due to conformational changes in subdomain 4, involving Thr203, Arg206, and seemingly originating from the bulky side chain of Tyr187, which is replaced by aspartate in canonical actins and glutamate in actin I, as discussed above. According to a recent filament structure [45], Tyr54 becomes stacked between the side chains of Asp52 and Lys62 of the same monomer and its only possible rotamers would allow an OH–π interaction with the phenyl ring of Tyr170 of the neighboring monomer. The side chain of Phe54 in actin I is not able to participate in this kind of an interaction due to the missing hydroxyl group. Thus, the replacement of tyrosine by phenylalanine at this position may affect both the rigidity of the D-loop in the monomeric state as well as the stability of the filament.

### Implications for catalysis

The state of the nucleotide-binding pocket can be described using two parameters: (i) the “phosphate clamp”, which is the distance between the Cα atoms of Gly15 and Asp157, and (ii) the “mouth”, which is the distance between the Cα atoms of Gln59 and Glu207 [46]. The phosphate clamp distance in *Plasmodium* actins does not differ significantly from other actins. However, the mouth of the binding pocket is significantly more closed in both parasite actins: 9.85 Å in actin I and 9.04 Å in actin II, compared to an average of 10.87±0.4 Å in 9 other actin–G1 structures used for comparison. In three recent high-resolution F-actin structures [28], [45], [48], the mouth distance varies between 7.88 and 9.66 Å. Both parasite actins reach the more closed conformation by slightly different means. In actin I, the largest differences in conformation to canonical actins are in subdomain 4 and in actin II, subdomain 2 (**Figs. 4** **and S3**).

**Figure 6:**
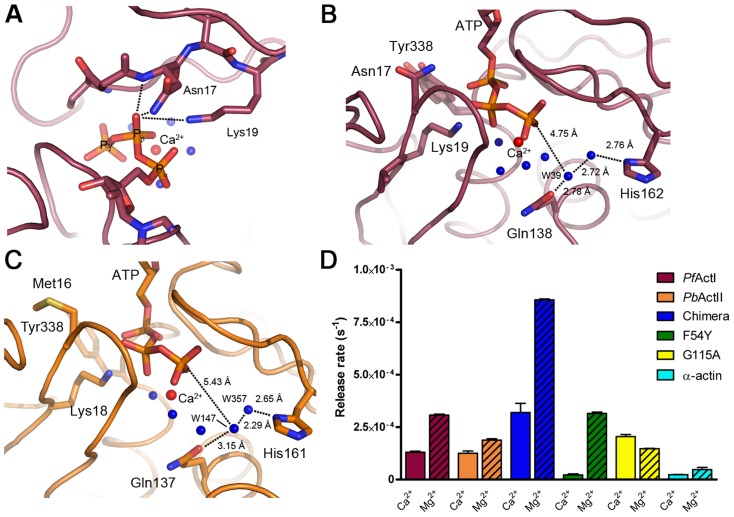
ATP binding sites of actin I and II. (**A**) The Asn17 side chain in actin I is part of a cluster formed by the Asn17 Nδ and main chain N atoms as well as Nζ of Lys19. Together, they could form an oxyanion hole for stabilizing a negative charge on one of the β-phosphate oxygen atoms in a reaction intermediate. (**B**) The active-site water structure in actin I is conserved, and W39 is in an almost inline position for a nucleophilic attack to the ATP γ-phosphate. (**C**) The catalytic water in actin II has moved further away from the ATP γ-phosphate, is mobile, and is likely a double conformation of the water bound directly to His161. (**D**) Phosphate release rates of the wild-type *Plasmodium* actins in the calcium- or magnesium-bound states compared to α-actin, the actin I–α-actin chimera and the actin I mutants F54Y and G115A. Error bars represent standard deviation (n = 3).

Both structures reported here have calcium and ATP bound to the nucleotide-binding cleft between subdomains 2 and 4 (**Figs. 4** **and** **6**). In the nucleotide-binding residues, there is one interesting difference in actin I compared to canonical actins and actin II; residue 17, which is hydrophobic (methionine/leucine) in all canonical actins and actin II, is an asparagine in actin I (**Fig. 6 A and B**) and also *T. gondii* actin. This side chain is close to the α- and β-phosphates of ATP. In actin I, the distance of the Asn17 Nδ2 atom to the β-phosphate O1 atom (∼3.75 Å) is too long for hydrogen bonding in this conformation. However, together with its own main chain N, that of Gly16, and Nζ of Lys19, the Asn17 side chain could form an oxyanion hole to stabilize a negative charge on the β-phosphate O1 atom (**Fig. 6 A**). In addition, it could interact with the α-phosphate. The Asn17 side chain is flexible in the crystal structure, as evident from the electron density maps and *B* factors, and based on the shape of the electron density as well as anisotropic ellipsoids, seems to move in concert with active-site water molecules as well as the nearby Tyr338, which is in a double conformation.

A catalytic mechanism based on a nucleophilic attack of a water molecule activated by His162 and Gln138 has been proposed for ATP hydrolysis in actin [57]. The reaction itself is rather simple, containing only proton transfer steps. The complication arises from the fact that G-actin – currently the only form of which atomic-resolution information can be achieved – is a poor catalyst, and conformational changes upon polymerization are needed for achieving the catalytically competent conformation of the active site. Water 39 (actin I numbering) has been proposed to be the nucleophile initiating the reaction, and depending on the bound metal, the position of this water changes [57]. In actin I, this water is 4.75 Å away from the γ-phosphate, and the angle between the β-γ bridging O, Pγ, and water 39 is 152.4°, which is amenable for a nucleophilic attack (**Fig. 6 B**). The catalytic site water structure in actin II is different compared to actin I (**Fig. 6 C**), as the presumed catalytic water (147 in the actin II structure) has moved towards His161 and Pro109 and has weak electron density and a high *B* factor. The distance of this water to Pγ is 5.43 Å, and it is in an almost in-line position (162.4°). In fact, according to the electron density and the distances to neighboring atoms, this water may be a second conformation of water 357, which is directly hydrogen bonded to His161. Together, these differences in the active site architectures between the two parasite actins and compared to other actins indicate that the catalytic activity and the exact mechanism of ATP hydrolysis may differ in them, likely resulting also in differences in polymerization.

Given the structural differences in the catalytic sites, we set out to test if the *Plasmodium* actins differ from each other and canonical actins in their ability to hydrolyze ATP and/or release phosphate. We first measured phosphate release in the presence of Mg^2+^ (**Fig. 6 D**). It should be noted that, as phosphate release at least in canonical actins is much slower than hydrolysis, this method gives only indirect information about the hydrolysis rate. In these conditions, both parasite actins release phosphate faster than α-actin. Of the two parasite actins, actin I has a slightly higher rate (∼2 times higher than actin II). Curiously, the chimera with the α-actin D-loop is approximately 3-fold more active than wild-type actin I and 23-fold more active than α-actin. We also made two point mutations to actin I; G115A, which we predicted to affect the flexibility of the proline-rich loop and, thus, the rate of hydrolysis, and F54Y, which we hypothesized might affect the rigidity of the D-loop in monomeric state. Neither of these mutants formed long filaments without JAS (**Fig. 1 F**). In the presence of Mg^2+^, the F54Y mutant shows identical behavior to wild-type actin I. G115A, however, has a reduced rate, similar to actin II. Also the kinetics differ from each other in the different actins. Whereas actin I and the point mutants release phosphate in a linear way, α-actin, actin II, and the chimera display more complex kinetics, having an initial very short, faster, non-linear phase, followed by a linear phase.

We next measured phosphate release in the Ca^2+^-bound, presumably mainly monomeric, forms (**Fig. 6 D**). As expected, α-actin showed an even lower release of phosphate in the Ca^2+^-bound compared to the Mg^2+^-bound form. Both actin I and actin II have activities equal compared to each other and approximately 5-fold higher than α-actin. The chimera releases phosphate 3-fold less in the presence of Ca^2+^ than with Mg^2+^, but the rate is still significantly higher than that of muscle actin or both wild-type parasite actins with Ca^2+^. Of the point mutants, F54Y has practically no activity with Ca^2+^ (identical to α-actin), whereas G115A is slightly more efficient in the presence of Ca^2+^ than Mg^2+^. Altogether, these data show that the *Plasmodium* actins have a different mechanism of ATP hydrolysis and/or subsequent phosphate release compared to canonical actins, which are poor catalysts in the monomeric form and adopt the catalytic conformation only upon polymerization, which is a prerequisite for non-equilibrium polymerization kinetics enabling directional growth [58]. We were also able to pinpoint amino acid residues responsible for these differences.

### *Plasmodium* actins have a unique response to ADP

**Figure 7:**
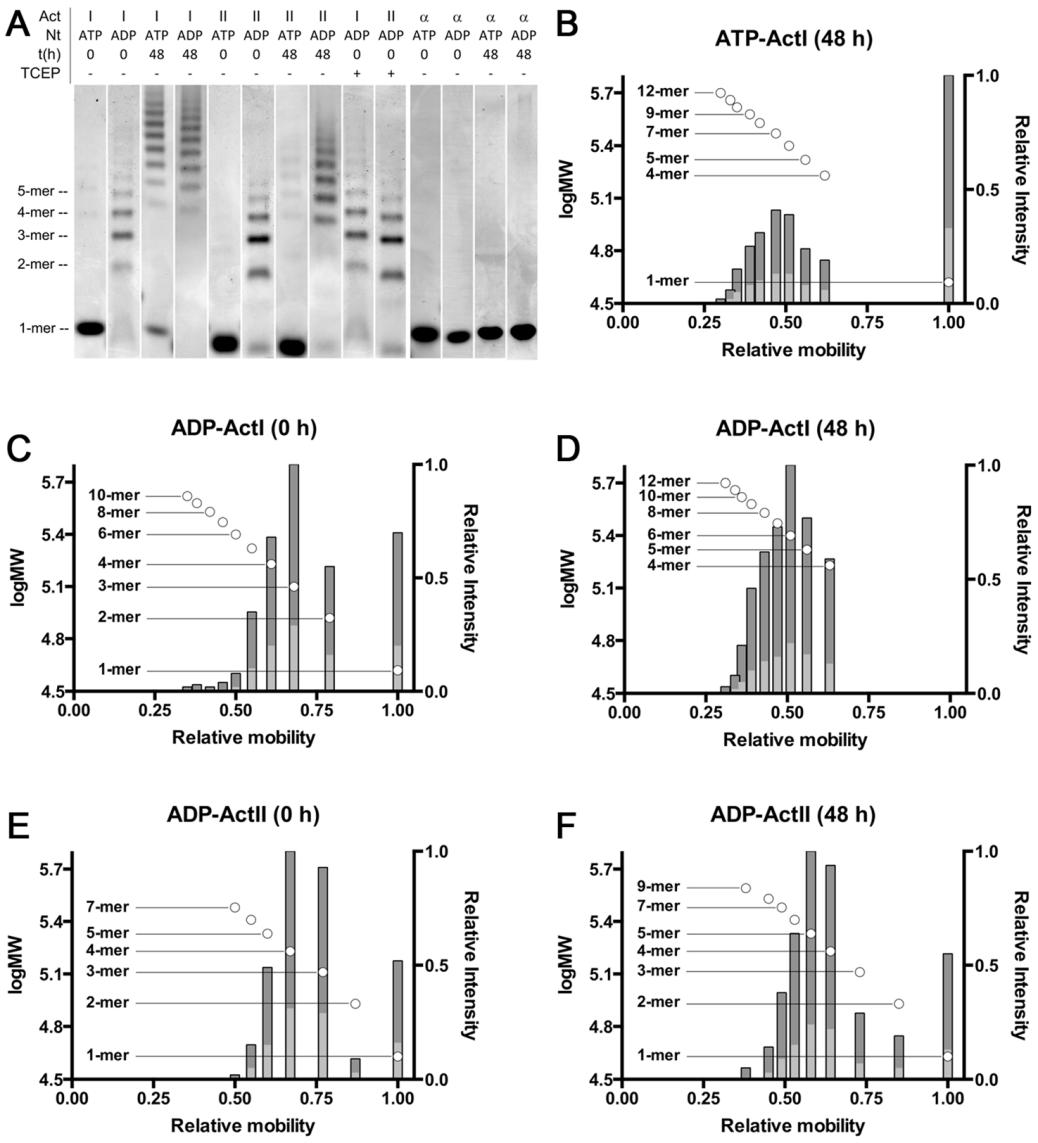
Native PAGE analysis of the *Plasmodium* actins and α-actin. (**A**) *Plasmodium* actins I (lanes 1–4 and 9) and II (lanes 5–8 and 10) form small oligomers upon storage and after exchange of ATP to ADP. Both parasite actins were studied by native PAGE immediately and 48 h after purification in both ATP and ADP forms. Treatment of ADP-exchanged *Plasmodium* actins with a high concentration of reducing agent (10 mM TCEP) has no effect on the behavior of either of the actins. Exchange of ATP to ADP in α-actin (lanes 11–14) does not result in changes in the oligomeric state. Nt denotes the nucleotide. TCEP - and + denote either the normal 1 mM or an excessive 10 mM concentration, respectively. The approximate position of the different oligomers, corresponding to lane 2, are given on the left. Note that actin I and II run slightly differently on the gel. (**B–F**) The relative mobility *vs.* log MW (circles with the oligomeric state indicated on the side) and relative intensities of bands (bars) extracted from gel images of Coomassie-stained native PAGE gels containing ATP or ADP *Plasmodium* actin I (**B–D**) and ADP actin II (**E and F**) immediately or 48 h after purification. The dark grey bars denote the relative intensity of the bands compared to the most intense band and the light grey bars the relative intensity of the bands compared to the sum of all band intensities.

In order to evaluate the oligomeric state of the parasite actins in the presence of ATP/ADP and different ions, we used native PAGE. With ATP bound, actin I spontaneously forms short polymers (from tetramers up to 11–12-mers) in ∼48 h when stored on ice (**Figs. 7** **and S5**). Actin II stays mainly monomeric over the same period of time, although minute amounts of oligomers (dimers–octamers) appear. Interestingly, in the presence of ADP, oligomerization starts instantly, and the majority of both actins is oligomeric (dimers–10-mers) immediately after a 1-h hexokinase treatment at 298 K to remove ATP. After incubation of the actins at 298 K for 1 h without hexokinase, only minute amounts of oligomers can be visualized for actin I, and no visible oligomerization of actin II takes place (data not shown). After 48 h, the proportion of larger oligomers of the ADP forms is much higher, and monomers as well as lower oligomers are practically non-existent. The formation of oligomers is not caused by oxidation, as using even a large excess of the reducing agent TCEP in the sample does not reduce the amount of oligomerization (**Fig. 7 A**). Both ATP and ADP forms of α-actin remain monomeric in the same conditions. However, short oligomers of ATP-α-actin have been reported below the critical concentration for polymerization [59].

Because the distribution, when separated on a gel, does not necessarily reflect the equilibrium between different species in solution, we also used dynamic light scattering (DLS) to visualize the size distribution and polydispersity of the actin mono- and oligomers in solution over time (**Fig. S6**). The resolution of DLS is far from that of the native gel assay, and it is only possible to detect size differences of approximately 5–6 fold. Therefore, *e.g.* monomers, dimers, and trimers will appear as a single, polydisperse peak. 6 h after purification, actin I is seen in mainly two separate peaks of average hydrodynamic radii of approximately 2 and 6 nm (**Fig. S6 A**). 2 nm would be very close to the expected hydrodynamic radius of the monomer. After 11 h, nearly all of actin I is in particles with a radius of ∼5 nm (**Fig. S6 B**). As time goes by, the distribution becomes divided between particles of below 3 nm (close to a monomer) and larger oligomers with an average radius of 11–12 nm (**Fig. S6 C and D**). The polydispersity of the sample after 11 h is very high, indicating that the sample contains a mixture of monomers and small oligomers, and the polydispersity diminishes again, as the sample gains a multimodal distribution, indicating that the smallest oligomers disappear over time, leaving behind a pool of monomers in addition to the higher oligomers, consistent with our native PAGE data. As seen also in the native gels, actin II retains a higher fraction of monomers over 48 h, but also gains a fraction of significantly higher oligomers, which are, however, infrequent and very heterogeneous in size (**Fig. S6 E–H**).

In order to probe the effects of Mg^2+^ and K^+^ ions on the oligomerization behavior, we also performed native PAGE in the presence of two concentrations (1 and 5 mM) of MgCl_2_ as well as 5 mM MgCl_2_ and 50 mM KCl (**Fig. S7**). In the presence of ATP, Mg^2+^ slightly reduces the amount of the short oligomers for both actin I and II compared to the Ca^2+^ forms (**Fig. S7 A and B**). However, some actin I is visible at the bottom of the well at the top of the gel, which would imply filaments too long to enter the gel. This could not be seen in the Ca^2+^ gels for either the *Plasmodium* proteins or α-actin, but was much more pronounced for α-actin with Mg^2+^. In the presence of ADP, there is a clear shift towards longer oligomers in actin I, and after 48 h, part of actin I stays in the well, not entering the gel in the presence of 5 mM MgCl_2_ both with and without KCl (**Fig. S7 C and D**). Thus, Mg^2+^ alone seems to be sufficient for polymerization.

### Actin I cannot replace actin II in male gametogenesis

We used genetically modified parasites to address the question whether the observed structural differences translate into different properties of the proteins *in vivo*. While it is not possible to delete actin I due to its essential functions, a knock-out of the *actin2* gene has been done, resulting in a block of male gametogenesis [23], [60]. We reasoned that a replacement of *actin2* with *actin1* would display the mutant phenotype if the two actin isoforms have different biological functions, while restoration of gametocyte development would indicate a similar function.

**Figure 8:**
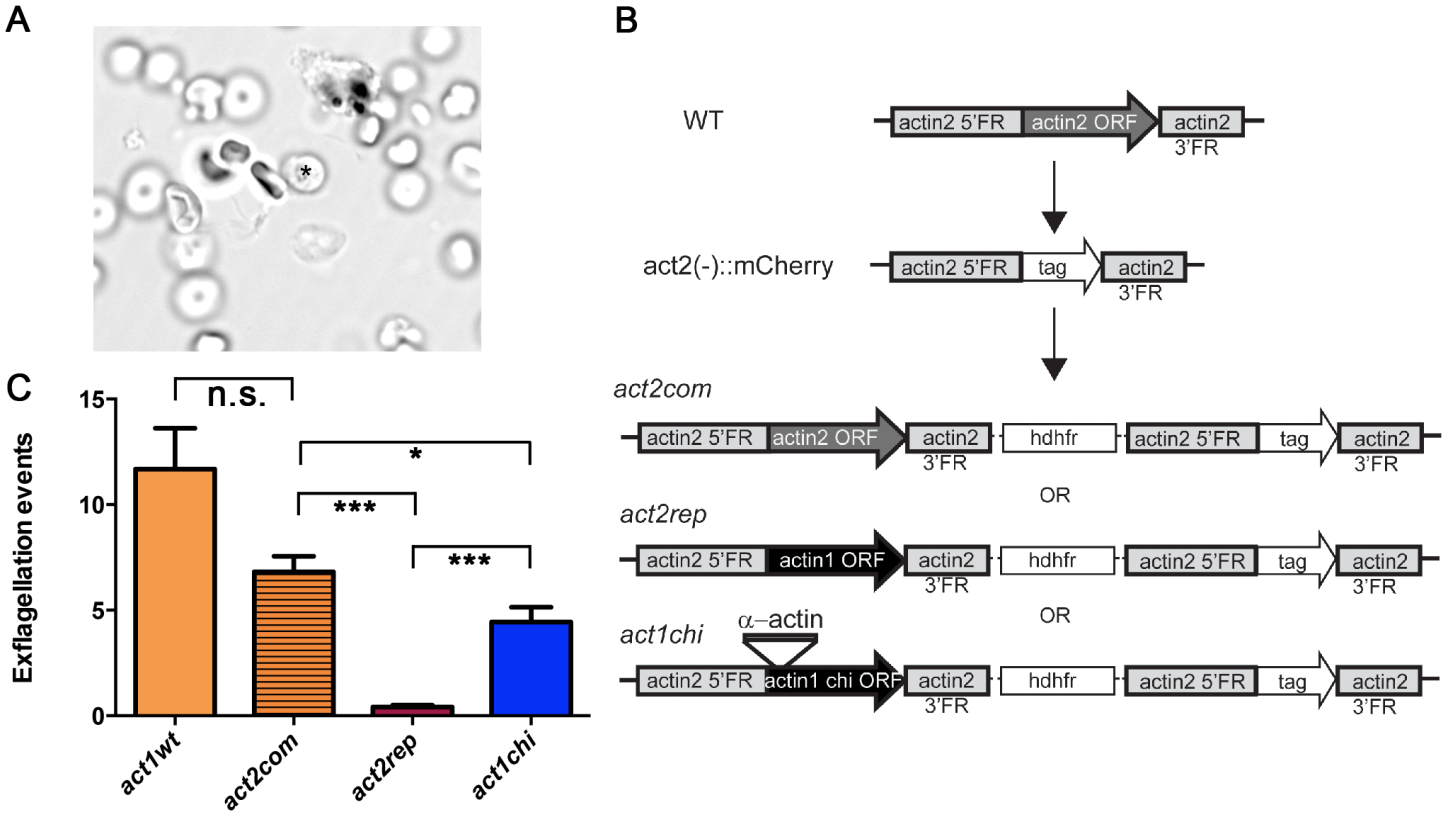
The D-loop chimera but not wild-type actin I rescues the phenotype of the *actin2* deletion mutant. (**A**) Exflagellation of a male gametocyte. The residual *P. berghei* gametocyte is indicated with an asterisk. The flagellar male gametes beat rapidly. The picture is the first frame of **Video S1**. (**B**) Schematic picture of the *actin2* gene in wild-type parasites, the recipient strain *act2^−^::mCherry*, and the final genotypes of the *act2com*, *act2rep*, and *act1chi* strains. The same experimental strategy was used for all constructs. The *act1chi* strain expresses a chimeric actin, where the D-loop (amino acids 39–61) has been swapped with the D-loop of α-actin (**C**) Exflagellation assays comparing wild-type (n = 11), *act2com* (n = 11), *act2rep* (n = 15) and *act1chi* (n = 14) parasites. The *act2com* functionally complemented the mutant, resulting in almost wild-type levels of exflagellation. The *act2rep* parasites had a significantly smaller number of exflagellation events, while swapping the D-loop of actin I with that of α-actin in the *act1chi* mutant results in significant restoration of exflagellation. n.s. means non significant, *** stands for P<0.0001, * for P<0.05 (Student's t-test).

Male gametogenesis in the malaria parasite is a unique event, involving the formation of flagellar gametes. This event, called exflagellation (**Fig. 8 A** **and Video S1**), is easily scored under the microscope, allowing us to use it as a quantitative method. In our approach, *actin1* was expressed under the control of the *actin2* flanking regions. We used a recipient line, in which the complete open reading frame (ORF) of *actin2* had been deleted. Therefore, these parasites do not exflagellate [60]. This line was separately transfected with two constructs, both aiming at integration into the *actin2* locus. The complementation construct (*act2com*) restored the *actin2* ORF, which allowed expression of the cognate gene comparable to wild type. In the replacement construct (*act2rep*), a fragment corresponding to the *actin1* ORF was used instead of the *actin2* ORF. The constructs were otherwise identical and were integrated in the locus *via* a single crossover homologous recombination event in the 5′ flanking region of *actin2* (**Fig. 8 B**). In both cases, clonal lines were obtained. We compared the *act2com* and *act2rep* parasite lines with wild-type parasites in the exflagellation assay (**Fig. 8 C**). In the wild-type and *act2com* parasites, the number of exflagellation events was similar, indicating that complementation with *actin2* restored the function of the gene. However, while some normal exflagellation events were detected also in the *act2rep* parasites, the numbers were significantly reduced compared to the *act2com* parasites (**Fig. 8 C**), strongly suggesting that actin II has unique functions, which actin I cannot fulfill during male gametogenesis.

### The actin I–α-actin D-loop chimera restores gametogenesis

As it became apparent that actin I polymerization properties *in vitro* could be altered by exchanging its D-loop to that of α-actin, we decided to investigate if this modification would also have an impact on the *in vivo* function of actin I. We produced transgenic parasites using the same strategy as described above; inserting the actin I–α-actin D-loop chimera into the *actin2* locus, producing the *act1chi* parasites (**Fig. 8 B**). Surprisingly, this revealed that the exchange of the D-loop had a remarkable impact on exflagellation (**Fig. 8 C**). In the *act1chi* parasites, exflagellation was significantly increased compared to the *act2rep* strain and restored to values close to the *act2com* strain. These data show that the D-loop has a critical role in the function of these actins, but actin II, as shown by the structural data, has acquired other properties that contribute to its higher filament stability. Furthermore, and to our surprise, it seems that the molecular function of actin II may be dependent on the ability of the protein to form filaments.

## Discussion

An actin cytoskeleton was long thought to be a feature unique to eukaryotic cells, and this view was revisited only two decades ago upon the discovery of the first bacterial actin and tubulin homologs [61]–[64]. The ancient phylum *Apicomplexa* is likely separated from opisthokonts by an evolutionary distance of a billion years, and the diversion of *Plasmodium spp.* took place hundreds of millions of years ago [65]. Therefore, looking at the divergent properties of *Plasmodium* actins provides us with insight into the early stages of actin evolution. The ability of actin to polymerize must have evolved very early – before its involvement as tracks for molecular motors [66]. This may explain some features of both the polymerization propensity and the divergent actin-myosin motor in *Apicomplexa*. Also the minimal set of actin-binding proteins in *Apicomplexa* suggests that a common ancestor had a limited polymerization propensity, and the various regulatory proteins in higher eukaryotes have evolved as the polymerization properties of actin itself have been fine-tuned, creating a need for additional regulation. For the biological functions of actin in *Apicomplexa*, the development of similar polymerization properties has strikingly not been of importance.

### Different polymerization properties provide evolutionary cues

The two *Plasmodium* actins differ in their polymerization propensities, filament stability, and filament helical symmetry – the hallmark of canonical F-actin. The second, stage-specific actin isoform of *Plasmodium* that forms long filaments with canonical F-actin symmetry is unique among *Apicomplexa*. Curiously, at the sequence level, actin II is as divergent from *Plasmodium* actin I as it is from all other actins. It has been suggested [23], and our structural data support the view, that the *actin2* gene has arisen only after the diversion of *Plasmodium* from other *Apicomplexa*, and the protein seems to have gained a higher filament stability independent of the evolution of higher eukaryotic actins.

In canonical actins, polymerization is tightly coupled to ATP hydrolysis, such that structural rearrangements upon polymerization enable the active site to adopt a conformation optimal for catalysis [67]. Two key factors have been described necessary for achieving the catalytically competent conformation upon the transition from monomeric to filamentous state. These are: (i) a rotation of the outer domain (subdomains 1 and 2), resulting in flattening of the monomer and (ii) bending down of the proline-rich loop in subdomain 1 [45], [48]. The *Plasmodium* actins hydrolyze ATP also in the monomeric form, releasing phosphate more efficiently than canonical actins, and oligomerize readily in the presence of ADP, which is a fundamental difference to all other actins characterized, and must be a result of different atomic structures.

Interestingly, *Plasmodium* actin I has a unique glycine at the end of the proline-rich loop. This allows more flexibility for this loop, which apparently increases the catalytic rate in the presence of magnesium but, surprisingly, has an opposite effect in the calcium-bound form (**Fig. 6 D**). Also the more closed conformations of subdomains 2 and 4 in the parasite actins may facilitate ATP hydrolysis but also reduce the conformational change – or flattening – required upon insertion of the monomer into the filament. An interesting difference that may also contribute to catalysis is Asn17 close to the α- and β-phosphates of ATP in the active site. Intriguingly, the bacterial actin homolog MreB [68] shares this residue with *Plasmodium* actin I, whereas canonical actins and also *Plasmodium* actin II have a hydrophobic residue at this position. Thus, this asparagine may be a relict from an early, polymerization incompetent ancestor.

The structural features described above may explain the parasite actins' unconventional response to ADP. Surprisingly, the state of the nucleotide seems to determine polymerization propensity, but not in the same way as in canonical actins. The tight link between ATP hydrolysis and polymerization in higher eukaryotes has probably been refined during the hundreds of millions of years after the diversion of *Apicomplexa*. Our data and a recent report proposing an isodesmic polymerization mode for apicomplexan actins [18] suggest that the same has also happened for allosteric regulation of conformational changes taking place upon polymerization. However, it is clear that higher resolution data on the *Plasmodium* actin filaments are needed in order to find out what kind of conformational changes the parasite actins undergo upon polymerization and what is the arrangement of the protomers in the filament, leading to the altered symmetry compared to canonical F-actin. On the other hand, the distribution of oligomers, as seen on the native gels and DLS (**Figs. 7** **, S5, and S6**), suggests that polymerization may involve a nucleation step, the nucleus being either a dimer or trimer, which are the species that disappear early in the process. Thus, we hypothesize that the ADP state may favor nucleation, making ATP hydrolysis a rate-limiting step for polymerization.

### Importance of the D-loop for polymerization

The D-loop plays a key role in the conformational changes upon polymerization as well as the conformation and stability of F-actin [28], [45], [47], [48]. Both previous work [26] and our EM analyses reveal differences in the helical architecture of actin I compared to α-actin. In the crystal structures, several regions important for intra-filament contacts in canonical actin filaments show substantial differences between the parasite and opisthokont actins, and the polymerization propensity and filament stability are overall likely a sum of numerous atomic details in the monomers. Yet, the sequence of the α-actin D-loop alone is sufficient to restore the ability of actin I to form long filaments, without altering the symmetry compared to the JAS-stabilized wild-type actin I filaments. Thus, whereas the longitudinal contacts by the D-loop are important for stability, the shape and symmetry of the filaments are determined by other factors.

Actin II shows us that stability can be obtained by other means than the D-loop, probably involving lateral interactions. In addition to the differences we have described in the residues involved in lateral contacts, a candidate responsible for increased stability is residue 200, which is a glycine in *Plasmodium* actin I and *T. gondii* actin [69] but serine or threonine in canonical actins as well as *Plasmodium* actin II and *Theileria* actin [9], all of which form long filaments. It has been reported that the double mutant G200S/K270M in *T. gondii* actin leads to an increased filament length when using phalloidin-labeled filaments [69]. However, we were not able to visualize long filaments of this mutant of *Plasmodium* actin I in polymerizing conditions without JAS (data not shown), indicating that several small changes are cumulatively responsible for the increased stability of actin II filaments.

The tip of the D-loop can adopt a helical conformation, albeit it is disordered in the vast majority of all G-actin structures, and appears mainly intrinsically disordered in solution in all nucleotide states of G-actin [70]. The likely higher helical propensity of the D-loop in *Plasmodium* actins may affect polymerization and filament stability in at least two different ways. If the helical conformation is more likely to occur in the filamentous form, this might actually facilitate polymerization, which would be in line with the proposed low critical concentration [15] or isodesmic model for polymerization of parasite actins [18]. However, it has also been proposed that the helical form occurs only transiently in the filament or that it is favored in the ADP form and leads to filament destabilization [54], [70]. In this way, a higher helical propensity would contribute to the lower stability of the parasite actin filaments.

Tyrosine hydrogen bonds can contribute substantially to protein stability [71]. Tyr54 is a phosphorylation target and plays a regulatory role in many actins [72]–[74]. For *Dictyostelium* actin, phosphorylation of this tyrosine increases the critical concentration and controls cell shape changes and spore formation [72]–[74]. In *Mimosa pudica L.*, a contact sensitive plant, where actin is heavily phosphorylated, tyrosine phosphatase inhibitors inhibit the fragmentation of actin filaments during leaf bending [75]. In addition to affecting binding to other proteins, phospho-Tyr54 stabilizes the D-loop conformation [74]. Upon polymerization, this region undergoes a large conformational change, and it seems that the OH group of Tyr54 may be involved in stabilizing interactions [45] that the Phe54 side chain could not fully compensate for. Only 11 of over 300 known actin sequences contain a phenylalanine at this position, and no other substitutions are known. Most of these 11 sequences are actins from *Plasmodium* or *Trypanosoma*, both species where actin filaments have not been observed *in vivo*. Despite the apparent importance of tyrosine at this position for normal actins, a single mutation to phenylalanine in *Dictyostelium* actin does not affect its polymerization properties [74]. In line with this, we also could not observe long filaments of the actin I F54Y mutant (**Fig. 1 F**). However, the large effect of the F54Y mutation on the phosphate release rate of actin I (**Fig. 6 D**) suggests that this residue, indeed, may significantly affect the conformation and flexibility of the D-loop.

Together, the above described structural properties may lead to a higher polymerization propensity but also lower filament stability in the parasite actins by lowering the energy barrier of the transition between monomeric and filamentous forms. Yet, the fact that the replacement of the D-loop alone is sufficient for stabilizing the filaments formed by actin I, while retaining their unique symmetry, is surprising, taking into account how similar the D-loops of the two *Plasmodium* actins with different stabilities are. This implies that, starting from an unstable filament forming ancestor, actin II has reached its present form mainly using other means than the D-loop for gaining additional filament stability.

### Roles of the two actins *in vivo*

Male gametogenesis is a complex, rapid series of cellular events including escape from the host cell, three mitotic divisions, and axoneme assembly, leading to the formation of eight flagellar and highly motile gametes from each gametocyte within 10–20 min from activation. Both actin isoforms are present in male gametocytes of *P. berghei* [23], but their function in these events is not understood. Actin II is not expressed in the asexual blood stages [23]. Its deletion blocks male gametogenesis, and therefore, these mutant parasites cannot be transmitted through the mosquito [23]. Still, it has not been possible to pinpoint the exact role of actin II. We show that the function of actin II cannot be complemented by actin I, proving distinct molecular functions for the two actins and suggesting that their unique structures and the differences in their ability to form filaments directly translate into different functional characteristics *in vivo*. By generating transgenic parasites expressing the actin I–α-actin chimera, we found that this mutant protein was able to function almost as well as actin II *in vivo*. This strongly confirms the *in vitro* experiments and supports the notion that the D-loop has a significant role in determining the polymerization properties of the parasite actins. Furthermore, we can hypothesize that the reason two actins evolved in *Plasmodium*, was the need to have actins with different propensities to polymerize in cells lacking a large repertoire of actin-binding proteins.

Another example of distinct general and reproductive actin isoforms can be found in plants, where it was recently shown that animal cytoplasmic but not muscle actins can take over the functions of the plant vegetative actins [76]. Remarkably, also three actins from single-celled protists could carry out the same tasks, suggesting that the properties required for fulfilling the cytoplasmic actin functions during spatial development in multicellular organisms were present already early on in the evolutionary history. However, it seems that the polymerization properties of both *Plasmodium* and all other actins have evolved separately, starting from a poorly polymerizing ancestor. It would be interesting to see if either of the *Plasmodium* actins can support spatial development in either plants or animals.

The current hypothesis is that actin I in *Plasmodium* is required for gliding motility, and the filaments involved need to be short and short-lived. Our data support this, as actin I forms only very short polymers. For the suggested role of actin I in gliding, the formation of long, stable filament seems undesirable [69]. Actin II clearly is able to form long filaments, which may be needed for functions specific to actin II within the mosquito stages, although such functions have not yet been specified. Intriguingly, *Plasmodium* appears to be the only apicomplexan parasite that has faced the evolutionary pressure for acquiring a second actin isoform that forms stable, long filaments.

### Concluding remarks

Our data provide a structural basis for understanding the different functional properties of the two actin isoforms of *Plasmodium spp.* These structures represent the, so far, most divergent and primitive actins characterized, and we show that the two isoforms have the most unique biochemical properties, structures, and biological functions of all known actin isoforms. High-resolution structural information will serve as a starting point for understanding these functions in detail and for evaluating the suitability of parasite actins and actin-binding proteins as drug targets.

## Materials and Methods

### Protein expression, purification, and biochemistry

Purification of G1 was performed as described [40]. Endogenous pig skeletal muscle α-actin was purified as described [36], [77]. *P. falciparum* actin I (PlasmoDB PF3D7_1246200) and *P. berghei* actin II (PlasmoDB PBANKA_103010) were expressed in *Sf*21 cells at 300 K, as described before [36]. A chimera, where residues 40–61 of the *P. berghei* actin I were replaced by the corresponding residues from α-actin, was cloned into pFastBac HT A (Invitrogen) and expressed in the same way as the wild-type actins. Two point mutations (G115A and F54Y) were introduced to actin I by incorporating the corresponding mutation to the 5′ end of the primers. The parental plasmid was cleaved with DpnI and recirculated with the T4 DNA ligase. The protein coding sequences were confirmed by DNA sequencing. The purification of the wild-type actin–G1 complexes was performed as described [40]. The chimera–G1 was also purified as described before for the two wild-type actins [40], except that HEPES (pH 7.5) was used in the lysis buffer, and size exclusion chromatography was performed in 10 mM HEPES (pH 7.5), 50 mM NaCl, 5 mM dithiothreitol (DTT), 0.2 mM CaCl_2_ and 0.5 mM ATP. Peak fractions containing the chimera–G1 were pooled and concentrated to 5.6 mg ml^−1^ for crystallization.

The purification of all the actin variants without G1 was performed essentially as described [40] except for a few modifications, as listed. For actin I, the lysis was carried out in 10 mM HEPES (pH 7.5), 5 mM CaCl_2_, 250 mM NaCl, 1 mM ATP, 5 mM β-mercaptoethanol, 15 mM imidazole, and size exclusion chromatography was performed in 15 mM HEPES (pH 7.0), 0.5 mM ATP, 5 mM DTT, and 0.2 mM CaCl_2_. The pH of the lysis buffer for actin II was 8.7, and size exclusion chromatography was performed in 25 mM Tris-HCl (pH 7.5), 0.5 mM ATP, 5 mM DTT, and 0.2 mM CaCl_2_. For the chimera, lysis was carried out in 20 mM HEPES (pH 7.5), 5 mM CaCl_2_, 250 mM NaCl, 1 mM ATP, 5 mM β-mercaptoethanol, 15 mM imidazole, and size exclusion chromatography was performed in 15 mM HEPES (pH 7.0), 0.5 mM ATP, 5 mM DTT, and 0.2 mM CaCl_2_. For DLS and filament length measurements, size exclusion chromatography was performed in 5 mM HEPES (pH 7.5), 0.5 mM ATP, 2 mM DTT, and 0.2 mM CaCl_2_. DLS was measured using a Wyatt DynaPro platereader-II and 15 or 30 µl of actin I and II at concentrations between 8.5–24 µM at 298 K. The measurements were performed in triplicate and the samples stored at room temperature between the measurements.

ADP-actin was prepared by incubating 50 µl of 10 µM actin with 1–2 mg of hexokinase-agarose beads (Sigma-Aldrich, #H-2653) in 15 mM HEPES pH 7.5, 1 mM ATP, 1 mM tris(2-carboxyethyl)phosphine (TCEP), 0.2 mM CaCl_2_, 2 mM D-glucose for 1 h at 298 K. As a control reaction, *Plasmodium* actins I and II were incubated in identical conditions without D-glucose and hexokinase and subsequently run on native PAGE. The residual ATP contamination in ADP stocks was removed by treating them in a similar fashion.

Native PAGE was performed using a running buffer of 25 mM Tris-HCl (pH 8.5), 195 mM glycine, 0.5 mM ATP or ADP, and 0.1 mM CaCl_2_ or MgCl_2_. The sample buffer consisted of 25 mM Tris-HCl (pH 8.5), 195 mM glycine, 10% (v/v) glycerol (final concentrations). Actin samples were loaded at a concentration of 6.7 µM in a volume of 10 µl. Commercial TGX 4–20% gradient gels (Biorad) were pre-run for 30 min at 277 K, 100 V before applying the samples. Samples were run for 7 h using the same voltage settings and temperature, with corresponding nucleotides and divalent cations in the running buffer. The gels were stained the next day with Coomassie Brilliant Blue R250.

Relative mobilities were determined by measuring the distance of the bands from the top of the image and dividing this value by that of the monomeric band. In the absence of a reference monomeric band in ADP-ActI (48 h), the absolute value from ATP-ActI (0 h) was used as a reference. The absolute mobilities of the other visible bands in these images had a difference of <2.5%. Gel images were processed and band intensities extracted using ImageJ [78]. A rolling ball background subtraction was applied before manually extracting the intensities.

Actin samples were prepared for the phosphate release assay by treating 10–15 µM purified actin with DOWEX 1X8 to remove nucleotides and free phosphate. After the removal of the nucleotide and phosphate, ATP was replenished by adding a small volume of a concentrated stock solution. Buffer controls were treated in a similar fashion, in order to reset the level of free phosphate and nucleotide compared to the samples. The concentration to be used for determining the release rate was measured from the nucleotide-free solutions in order to reduce the effect of pipetting errors. After the DOWEX treatment, samples were divided in triplicate wells of a UV-transparent 96-well plate (Corning) containing reagents from the EnzChek Phosphate Release Assay (Molecular Probes) without using the reaction buffer, which contains MgCl_2_ at a final concentration of 1 mM. For calcium measurements, the final reaction contained 1 mM CaCl_2_ and 0.1 mM MgCl_2_. The total omission of MgCl_2_ was not possible, since the coupled enzyme requires magnesium. For magnesium measurements, the respective concentrations were 0.13 mM CaCl_2_ and 1 mM MgCl_2_. Formation of the 2-amino-6-mercapto-7-methylpurine from the coupled reaction was measured as absorbance at 360 nm with a kinetic interval of 60 s over a period of 5 h at 298 K. The total measurement volume was 200 µl. Phosphate release rates were calculated from linear parts of the plot (100 to 200 min) using GraphPad PRISM 5.03.

### Crystallization, diffraction data collection, structure determination, and refinement

Crystallization and diffraction data collection of both wild-type actin–G1 complexes has been described [40]. The chimera–G1 complex was crystallized similarly, and the final crystallization condition contained 100 mM Tris-HCl (pH 8.0), 8% (w/v) polyethylene glycol (PEG) 20 000, and 2% (v/v) dioxane. Before flash-cooling in liquid nitrogen, the crystal was shortly soaked in 100 mM Tris-HCl (pH 8.5), 14% (w/v) PEG 20 000, 2% (v/v) dioxane, 0.5 mM ATP, 50 mM NaCl, 0.2 mM CaCl_2_, and 10% (w/v) PEG 400. A diffraction data set to 2.5-Å resolution was collected on a Pilatus 6M detector at the beamline P11, PETRA III (DESY), Hamburg, using a wavelength of 0.92 Å at 100 K. The data (**Table 1**) were integrated with XDS [79] and scaled with XSCALE [79] using XDSi [80]. The actin II–G1 structure was solved by molecular replacement with Phaser [81] using the α-actin–G1 complex as a search model (PDB code 1P8Z [82]). For actin I–G1 and chimera–G1, the actin II and actin I in complex with gelsolin, respectively, were used as molecular replacement models. The refinement was carried out with PHENIX.refine [83] and manual model building in Coot [84], and structure validation using the MOLPROBITY server [85]. For actin I, actin II, and chimera–G1 complexes, 99.8%, 99.8%, and 99.4% of the amino acids, respectively, were in the allowed regions of the Ramachandran plot. The final electron density maps as well as data and refinement statistics are presented in **Fig, S2 A–C** and **Table 1**. The structure figures were prepared using PyMOL and Chimera [86].

**Table 1:** Data collection and refinement statistics.

	Actin I–G1	Actin II–G1	Chimera–G1
**Data collection** *			
Space group	P2_1_2_1_2	P2_1_	P2_1_2_1_2_1_
Cell dimensions			
*a*, *b*, *c* (Å)	40.34, 57.90, 11.59	64.25, 60.91, 75.52	54.24, 69.53, 178.83
α, β, γ (°)	90, 90, 90	90, 97.24, 90	90, 90, 90
Resolution (Å)	45–1.19 (1.25–1.19)	31.9–2.20 (2.25–2.20)	40–2.5 (2.6–2.50)
*R_meas_* #	0.126 (1.194)	0.156 (0.786)	0.157 (1.601)
*CC_1/2_* †	0.999 (0.383)	0.986 (0.568)	0.998 (0.365)
*〈I*/σ(*I)〉*	10.9 (1.07)	5.6 (0.9)	9.2 (0.9)
Completeness (%)	100 (100)	99.4 (95.2)	99.6 (96.9)
Redundancy	7.2 (6.8)	3.6 (2.3)	6.7 (4.1)
**Refinement** *			
Resolution (Å)	55.2–1.30 (1.35–1.30)	31.91–2.20 (2.28–2.20)	38.58–2.50 (2.60–2.50)
No. reflections	134,201 (13,159)	29,446 (2,810)	22,903 (2,454)
*R*_work_/*R*_free_	0.121 (0.230)/0.154 (0.263)	0.196 (0.255)/0.228 (0.270)	0.211 (0.412)/0.264 (0.459)
No. atoms			
Protein	4,417	3,940	3,860
Ligand/ion	34	50	40
Water	774	348	56
*B* factors (Å^2^)			
Protein	15.6	36.2	75.0
Ligand/ion	9.7	41.0	69.5
Water	20.6	39.9	51.6
rms deviations			
Bond lengths (Å)	0.011	0.002	0.003
Bond angles (°)	1.45	0.67	0.68

*Values in parentheses are for the highest-resolution shell.

# R_meas_ is the redundancy-independent *R* factor [94], [95].

† CC_1/2_ is defined as the correlation coefficient between two random half data sets [96].

### Electron microscopy

Actin (7–13 µM) was polymerized overnight at room temperature. Polymerization was induced by adding 1/10 volume of 10× polymerization buffer [50 mM Tris-HCl (pH 8.0) or HEPES (pH 7.5), 500 mM KCl, 20 mM MgCl_2_ (in cryo-EM 40 mM MgCl_2_), 50 mM DTT, and 10 mM ATP] with or without 5–7 µM JAS. In order to concentrate the filaments, actin II and the chimera in F-buffer were spun for 45 min at 435,000 g, and remaining pellet was resuspended into polymerization buffer. 2–3-µl aliquots of the samples were diluted in the polymerization buffer before applying them on glow-discharged grids (CF-300CU, Electron Microscopy Sciences) and stained with 1% (w/v) uranyl acetate or potassium phospho-tungstate (pH 7.0). The grids were examined with Tecnai G2 Spirit (100 kV) or FEI Tecnai F20 microscopes (200 kV). Filament lengths were measured using ImageJ [78]. Many of the longest (>1 µm) measured filaments are fragments, as both ends were not always visible in the images.

### Electron cryo-microscopy

Polymerized samples were applied in 3-µl aliquots onto freshly glow-discharged holey carbon grids (Quantifoil R 2/2) at 295 K and 70% humidity and vitrified in liquid ethane using a Leica EM GP vitrification robot. Specimens were held in a Gatan 626 cryoholder maintained at 93 K for imaging in a FEI Tecnai F20 microscope operated at 200 kV. Micrographs were recorded under low dose conditions on a Gatan Ultrascan 4000 CCD camera at a magnification of 69,000 to give a final pixel size of 2.21 Å.

### Image processing

The contrast transfer function (CTF) of the micrographs was determined using CTFFIND [87]. A total of 330 (actin I), 56 (actin II) and 457 (chimera) filaments were selected using e2helixboxer.py from the EMAN2 suite [88]. For classification, segments were excised using a mean step size of 30 Å and an additional random shift along the helix between -15 and 15 Å to avoid high-resolution artifacts in the class average power spectra introduced by regularly shifted images. The segments were further corrected for their CTF by phase flipping, and aligned to the vertical axis. This resulted in 4,581 segments for actin I, 968 for actin II, and 8,052 for the chimera actin. Two-dimensional (2D) classification of helical segments was performed using the SPARX k-means algorithm [27]. The segments were iteratively classified and aligned against a subset of class-averages chosen based on their quality with a total of four iterations. At each cycle, multiple copies of the chosen references were created by applying integer y-shifts ranging from −15 Å to +15 Å in order to be able to reduce the Y-shift search range during alignment to less than half of the step size in order to avoid summation of successive images on a filament shifted at the same axial position. The total number of class averages used to measure the cross-over distance was 40 for actin I and the chimera actin, and 20 for actin II. In addition, Eigen images were calculated and the corresponding pitch distances were measured. For 3D structure determination of actin I filaments, 2,182 segments were excised using a regular step size of 70 Å, convolved by their respective CTF and further reconstructed as described [30] using the software SPRING [89]. In addition, symmetry refinement was performed using the IHRSR method [90] by systematically varying the initial helical rises and azimuthal rotations from 26 to 30 Å (step 1 Å) and from 164 to 170° (step 1°), respectively. More specifically, 25 iterations of refinement were computed with SPIDER, using a solid cylinder of 100 Å in diameter as a starting model. The symmetry parameters were refined with the *hsearch* program after the second refinement iteration, using a step size of 0.03 Å for helical rise and of 0.05° for azimuthal rotation.

### Genetic replacement of actin II with actin I in *Plasmodium berghei*

The *actin2* complementation and replacement constructs were made in a derivative of the pL0006 vector, which encodes human DHFR conferring resistance to the drug WR99210 [91], [92]. The design of the constructs is described in detail elsewhere [24], and the three different constructs were produced following the same strategy. Briefly, 2.7 kilobase pairs of the promoter and 728 base pairs of the 3′-flanking region of the *P. berghei actin2* gene were amplified from gDNA and cloned into the vector. For the *act2rep* construct, *P. bergei actin I* complete ORF including start and stop codon was amplified from gDNA and cloned between the *actin2* promoter and the 3′ flanking region of *actin2*. The same strategy was followed for the *act2com* construct using the *P. berghei actin2* ORF and the *act1chi* construct. The plasmids were linearized before transfection of the recipient *act2^−^::mCherry* parasite line [60]. Parasites were cloned as described [93]. Correct integration was verified by PCR genotyping and Southern blotting. Exflagellation was scored after diluting blood from an infected mouse in exflagellation medium [23] and incubating the samples for 10–20 min at 292 K. The exflagellation events were counted under a light microscope.

### Accession numbers

The structure factors and coordinates for all three crystal structures have been submitted to the PDB under the codes 4cbu, 4cbw, and 4cbx. The actin I EM map has been deposited to the EMDB under the accession code EMD-2572.

### Online supplemental material

**Figure S1** shows an alignment of apicomplexan and canonical actin sequences. **Figure S2** shows the electron density maps around the ATP-binding site of the *Plasmodium* actins and the chimera and the gelsolin complexes for actin I and II in two orientations. **Figure S3** depicts root mean square deviations between *Plasmodium* and canonical actin structures. **Figure S4** shows the cryo-EM analysis of the actin I–α-actin chimera filaments. **Figure S5** shows native PAGE analysis of the *Plasmodium* actins in the calcium-bound form. **Figure S6** shows the DLS analysis of the oligomerization of the parasite actins over time. **Figure S7** shows native gels of the *Plasmodium* actins in the magnesium-bound form. **Video S1** shows an exflagellation event of a male *P. berghei* gametocyte.

## Supporting Information

Figure S1 **Sequence alignment of selected apicomplexan and canonical actins.** The following sequences were used for the alignment: *P. falciparum* actin I (*Pf*ActI), *P. berghei* actin II (*Pb*ActII), *T. gondii* actin (*Tg*Act), *Dictyostelium discoideum* actin (*Dd*Act), *Saccharomyces cerevisiae* actin (*Sc*Act), *Arabidopsis thaliana* actin (*At*Act), *Homo sapiens* skeletal muscle α-actin (*Hs*Act_alpha_sk), *H. sapiens* cytoplasmic β-actin (*Hs*Act_beta_cp), and *H. sapiens* smooth muscle γ-actin (*Hs*Act_gamma_sm). The numbering refers to, and the secondary structure assignment is based on, *P. falciparum* actin I. The black coils indicate α-helices and black arrows β-strands. Residues identical in all sequences are colored blue, and residues in red boxes are either identical or have similar properties. The yellow, pink, and orange highlights denote the D-loop (residues 39–61), the proline-rich loop (residues 109–115), and the hydrophobic loop (residues 263–275), respectively. Residues marked with blue and green stars are those discussed in the text as being involved in intra-protofilament or inter-protofilament contacts, respectively. Cyan stars indicate residues implicated in catalysis and pink ones those discussed in the context of Tyr54 in the D-loop. Trp357 in the hydrophobic cleft is indicated by a black star, the hinge region (prolines 333–334) with orange stars, and Ser366, where the C terminus makes a turn in actin I by a red triangle.
(PDF)

Figure S2 **Quality of the electron density maps around the ATP-binding sites and visualization of G1 binding to the** ***Plasmodium*** **actins.** (**A**) Actin I–G1, (**B**) actin II–G1, and (**C**) chimera–G1. The electron density is contoured at 2 σ. ATP and surrounding residues are labeled. (**D**) Cartoon representation of actin I in complex with G1 (red) superimposed on an α-actin-G1 complex (cyan; 1eqy [39]). Actin is above, gelsolin below, as indicated. The right-hand panel is rotated by 90° compared to the left panel. The hydrophobic loop is indicated in the right panel. (**E**) Cartoon representation of actin II in complex with G1 (orange) superimposed on α-actin (cyan). The orientation and labeling are as in (**D**).
(TIF)

Figure S3 **Root mean square deviations (rmsd) between** ***Plasmodium*** **actin and canonical actin structures.** The structures were superimposed using the Matchmaker tool in Chimera [86]. The gray-colored ribbons have been excluded from the rmsd calculation. The color panel below presents the rmsd, which is also highlighted with the thickness of the ribbon. The *Plasmodium* actin structures are compared against each other and canonical muscle and non-muscle actin–G1 complexes (1eqy [39]; rabbit α-actin–G1 and 3cip [72]; *Dictyostelium discoideum* actin–G1).
(TIF)

Figure S4 **Summary of the symmetry analysis of the actin I–α-actin chimera filaments.** (A) Filaments embedded in vitreous ice. (B) Histogram of half-pitch distances from measurements of (C) class averages. (D) Eigen images. (E) Symmetry analysis.
(TIF)

Figure S5 **Native PAGE analysis.** The relative mobility *vs.* log MW (circles) and relative intensities of bands (bars) extracted from gel images of Coomassie-stained native gels containing the ATP forms of *Plasmodium* actin I immediately after purification (**A**) and actin II 0 and 48 h after purification (**B,C**). The dark grey bars denote the relative intensity of the bands compared to the most intense band, and the light grey bars the relative intensity of the bands compared to the sum of all band intensities.
(TIF)

Figure S6 **Hydrodynamic radii and polydispersity of actins I and II over time as measured by dynamic light scattering.** (**A–D**) *P. falciparum* actin I. (**E–H**) *P. berghei* actin II. The average hydrodynamic radius (in nm) of each species and its standard deviation (n = 3) are shown close to the bars in all panels. The larger particles, for which no standard deviations are given, were only observed in one of the triplicate measurements and represent only a very small fraction of the total mass. Note the different (and logarithmic) scale of the X axis in (**A–D**) compared to (**E–H**).
(TIF)

Figure S7 **Behavior of** ***Plasmodium*** **actins and α-actin in native PAGE in the presence of magnesium and potassium.** Native PAGE gels showing *Plasmodium* actins and α-actin in ATP (**A, C**) and ADP (**B, D**) forms 0 and 48 h after purification with either 1 mM MgCl_2_, 5 mM MgCl_2_ and 0.5 mM EGTA, or 5 mM MgCl_2_, 0.5 mM EGTA, and 50 mM KCl in the sample.
(TIF)

Video S1 **Exflagellation of a male gametocyte.** The flagellar *P. berghei* male gametes are seen beating rapidly. Images were recorded using a light microscope at 1 frame/s, and the video is playing at 5 frames/s.
(AVI)
