# Supplementary figures and images for: Structural Differences Explain Diverse Functions of Plasmodium Actins
Source: PLoS Pathog. 2014 Apr 17;10(4):e1004091. doi: 10.1371/journal.ppat.1004091 (PMC3990709; doi:10.1371/journal.ppat.1004091)

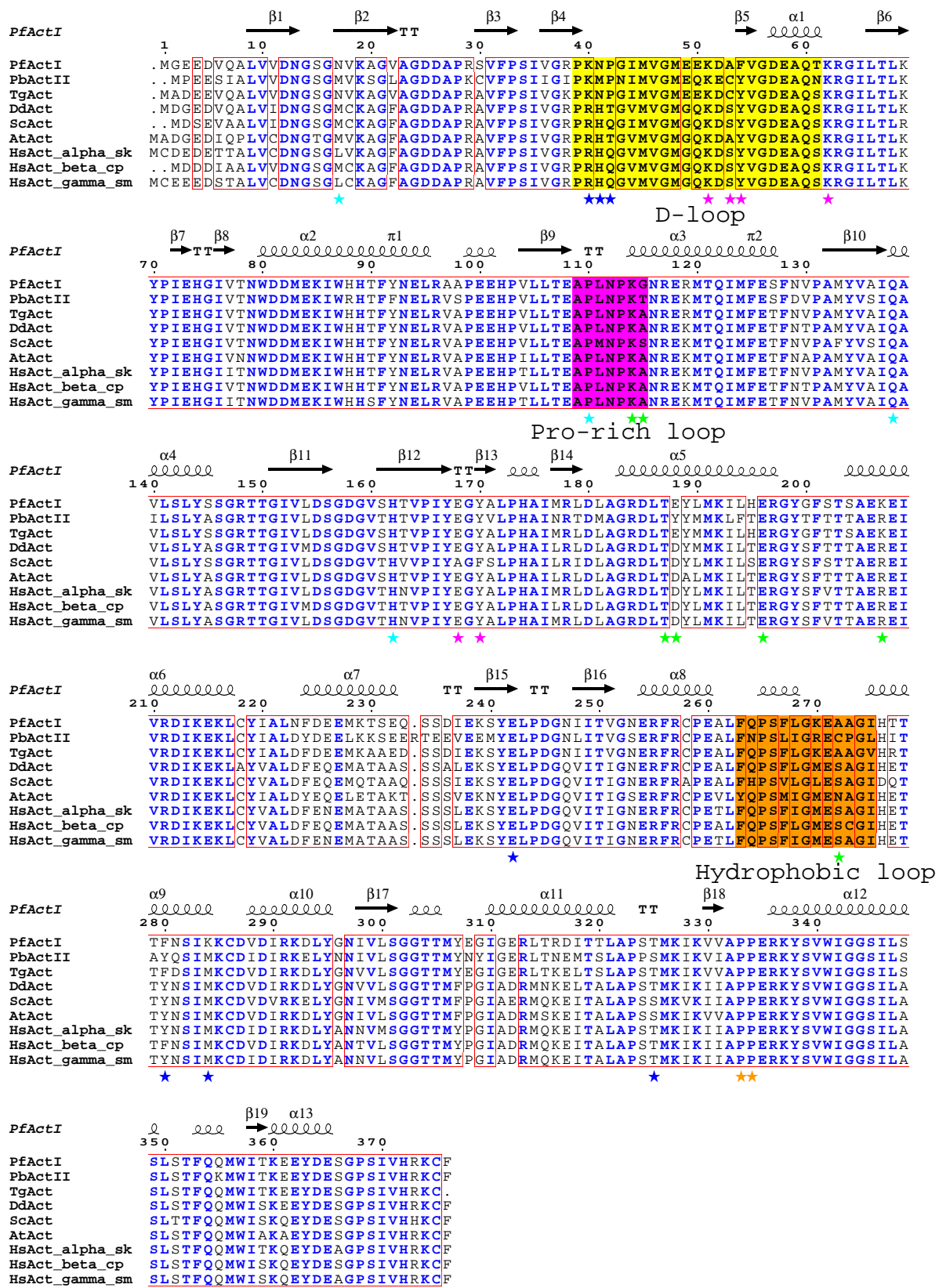

Supplementary Fig. 1 - Kursula

Supplement: Figure S1 — Sequence alignment of selected apicomplexan and canonical actins. The following sequences were used for the alignment: P. falciparum actin I (PfActI), P. berghei actin II (PbActII), T. gondii actin (TgAct), Dictyostelium discoideum actin (DdAct), Saccharomyces cerevisiae actin (ScAct), Arabidopsis thaliana actin (AtAct), Homo sapiens skeletal muscle α-actin (HsAct_alpha_sk), H. sapiens cytoplasmic β-actin (HsAct_beta_cp), and H. sapiens smooth muscle γ-actin (HsAct_gamma_sm). The numbering refers to, and the secondary structure assignment is based on, P. falciparum actin I. The black coils indicate α-helices and black arrows β-strands. Residues identical in all sequences are colored blue, and residues in red boxes are either identical or have similar properties. The yellow, pink, and orange highlights denote the D-loop (residues 39–61), the proline-rich loop (residues 109–115), and the hydrophobic loop (residues 263–275), respectively. Residues marked with blue and green stars are those discussed in the text as being involved in intra-protofilament or inter-protofilament contacts, respectively. Cyan stars indicate residues implicated in catalysis and pink ones those discussed in the context of Tyr54 in the D-loop. Trp357 in the hydrophobic cleft is indicated by a black star, the hinge region (prolines 333–334) with orange stars, and Ser366, where the C terminus makes a turn in actin I by a red triangle. (PDF) [file ppat.1004091.s001.pdf]

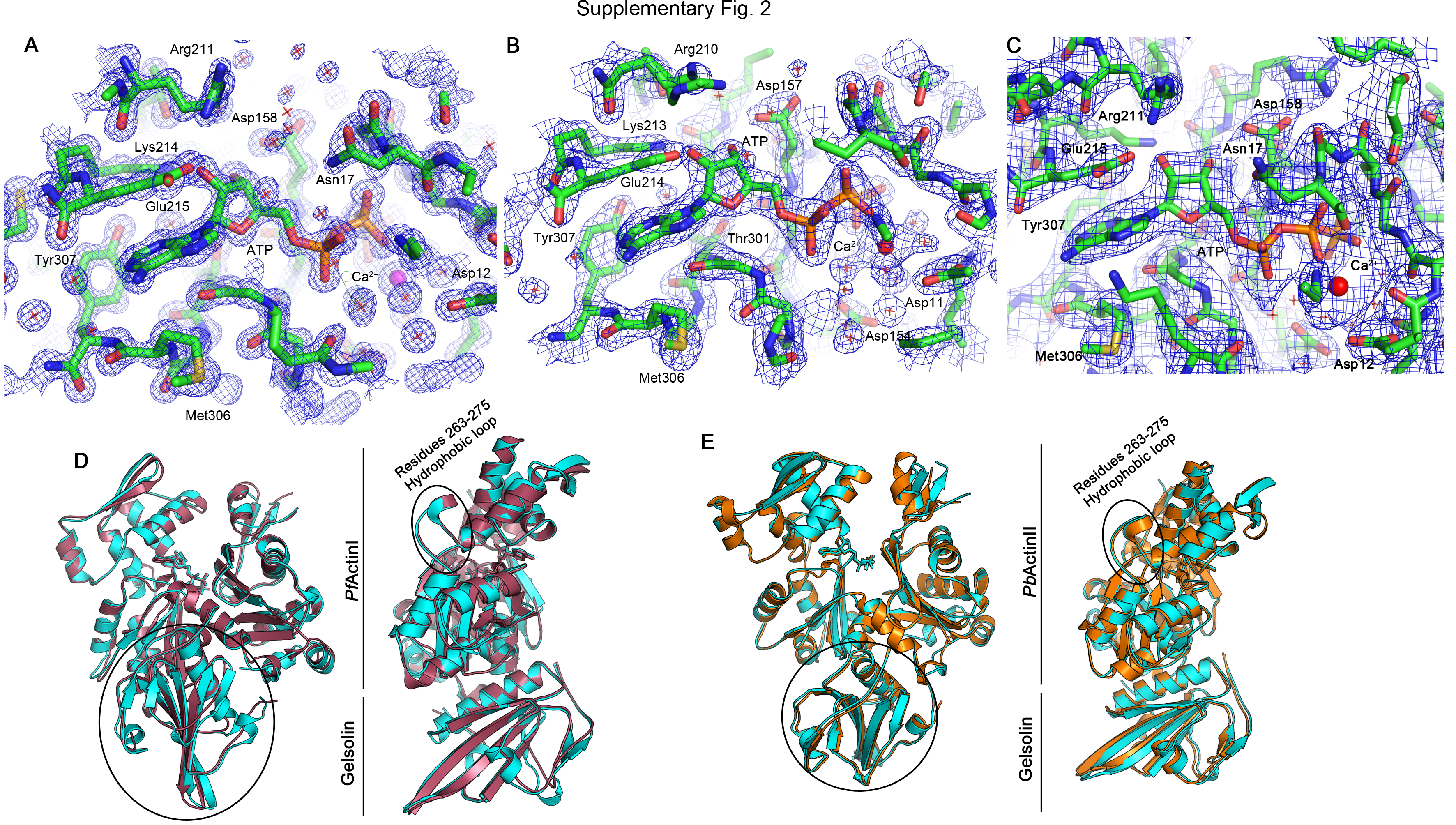

Supplement: Figure S2 — Quality of the electron density maps around the ATP-binding sites and visualization of G1 binding to the Plasmodium actins. (A) Actin I–G1, (B) actin II–G1, and (C) chimera–G1. The electron density is contoured at 2 σ. ATP and surrounding residues are labeled. (D) Cartoon representation of actin I in complex with G1 (red) superimposed on an α-actin-G1 complex (cyan; 1eqy [39]). Actin is above, gelsolin below, as indicated. The right-hand panel is rotated by 90° compared to the left panel. The hydrophobic loop is indicated in the right panel. (E) Cartoon representation of actin II in complex with G1 (orange) superimposed on α-actin (cyan). The orientation and labeling are as in (D). (TIF) [file ppat.1004091.s002.tif]

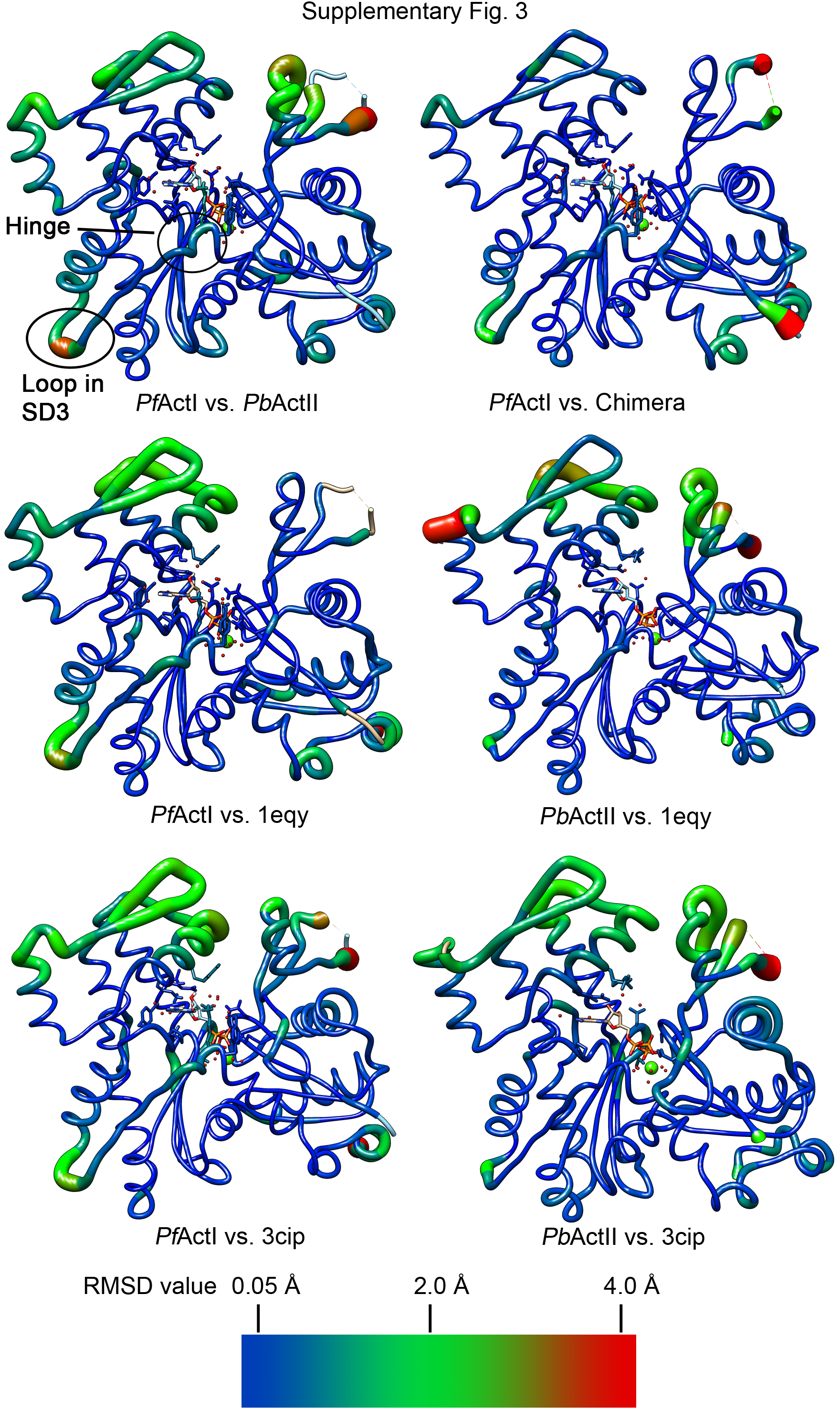

Supplement: Figure S3 — Root mean square deviations (rmsd) between Plasmodium actin and canonical actin structures. The structures were superimposed using the Matchmaker tool in Chimera [86]. The gray-colored ribbons have been excluded from the rmsd calculation. The color panel below presents the rmsd, which is also highlighted with the thickness of the ribbon. The Plasmodium actin structures are compared against each other and canonical muscle and non-muscle actin–G1 complexes (1eqy [39]; rabbit α-actin–G1 and 3cip [72]; Dictyostelium discoideum actin–G1). (TIF) [file ppat.1004091.s003.tif]

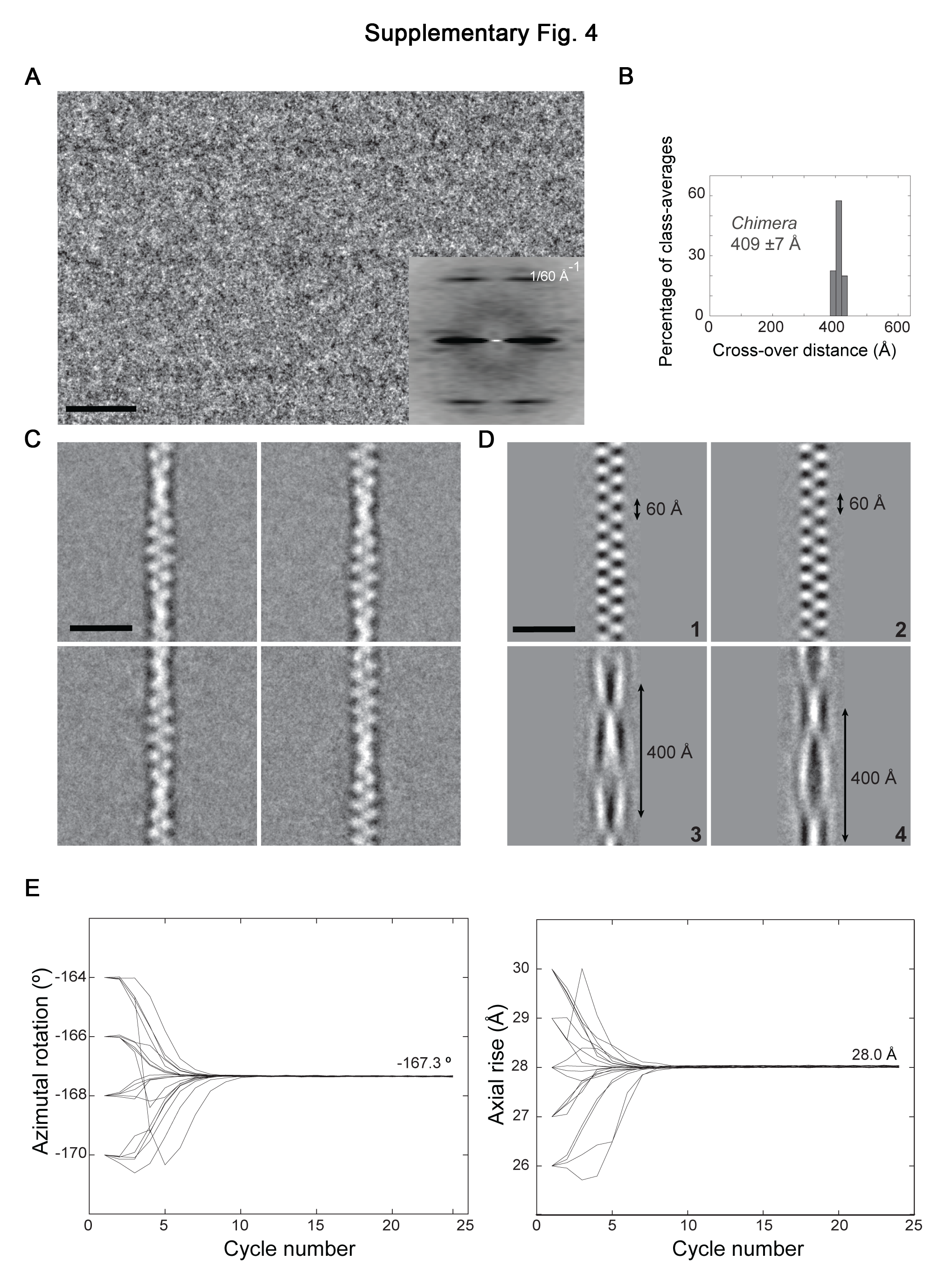

Supplement: Figure S4 — Summary of the symmetry analysis of the actin I–α-actin chimera filaments. (A) Filaments embedded in vitreous ice. (B) Histogram of half-pitch distances from measurements of (C) class averages. (D) Eigen images. (E) Symmetry analysis. (TIF) [file ppat.1004091.s004.tif]

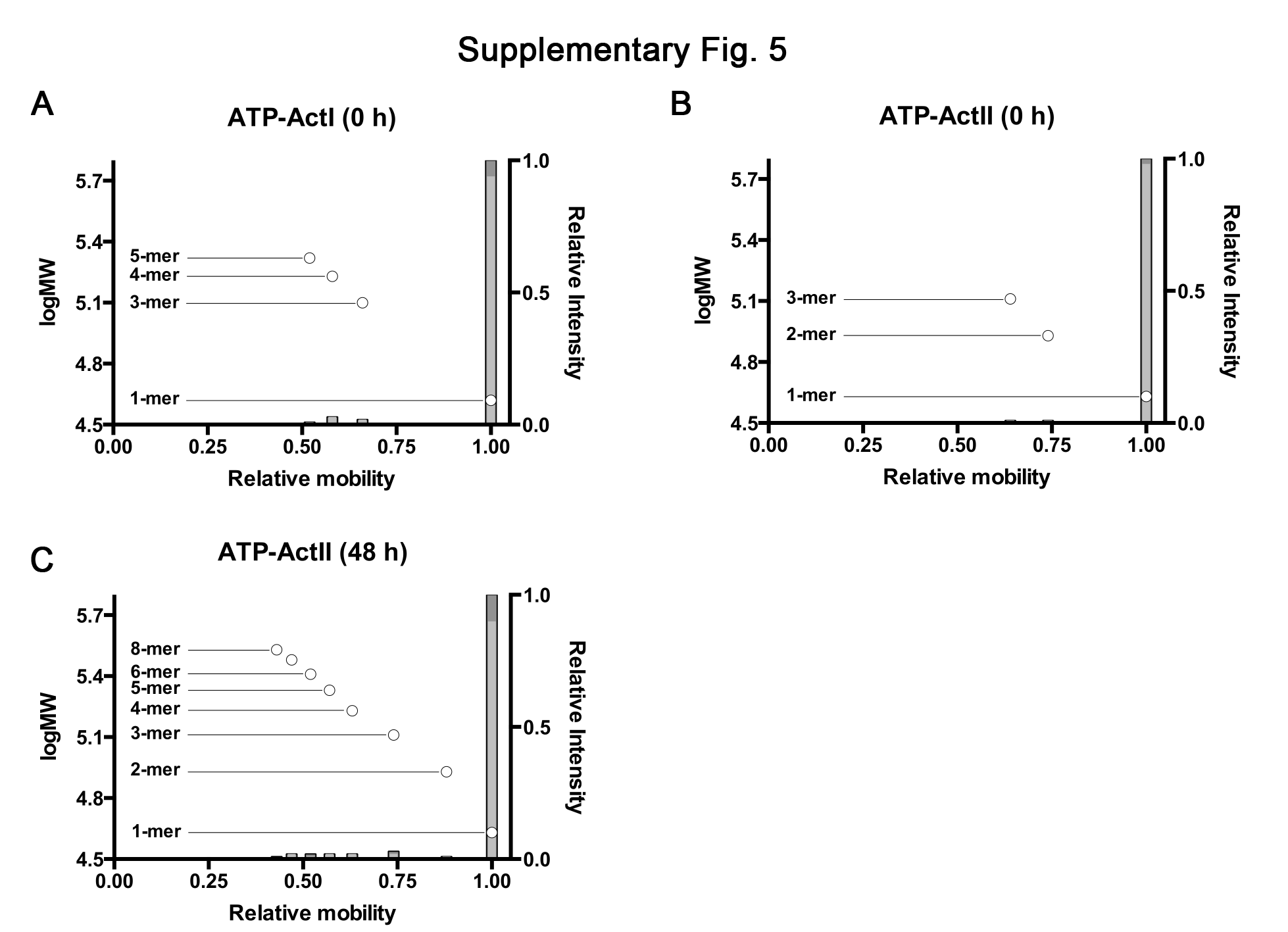

Supplement: Figure S5 — Native PAGE analysis. The relative mobility vs. log MW (circles) and relative intensities of bands (bars) extracted from gel images of Coomassie-stained native gels containing the ATP forms of Plasmodium actin I immediately after purification (A) and actin II 0 and 48 h after purification (B,C). The dark grey bars denote the relative intensity of the bands compared to the most intense band, and the light grey bars the relative intensity of the bands compared to the sum of all band intensities. (TIF) [file ppat.1004091.s005.tif]

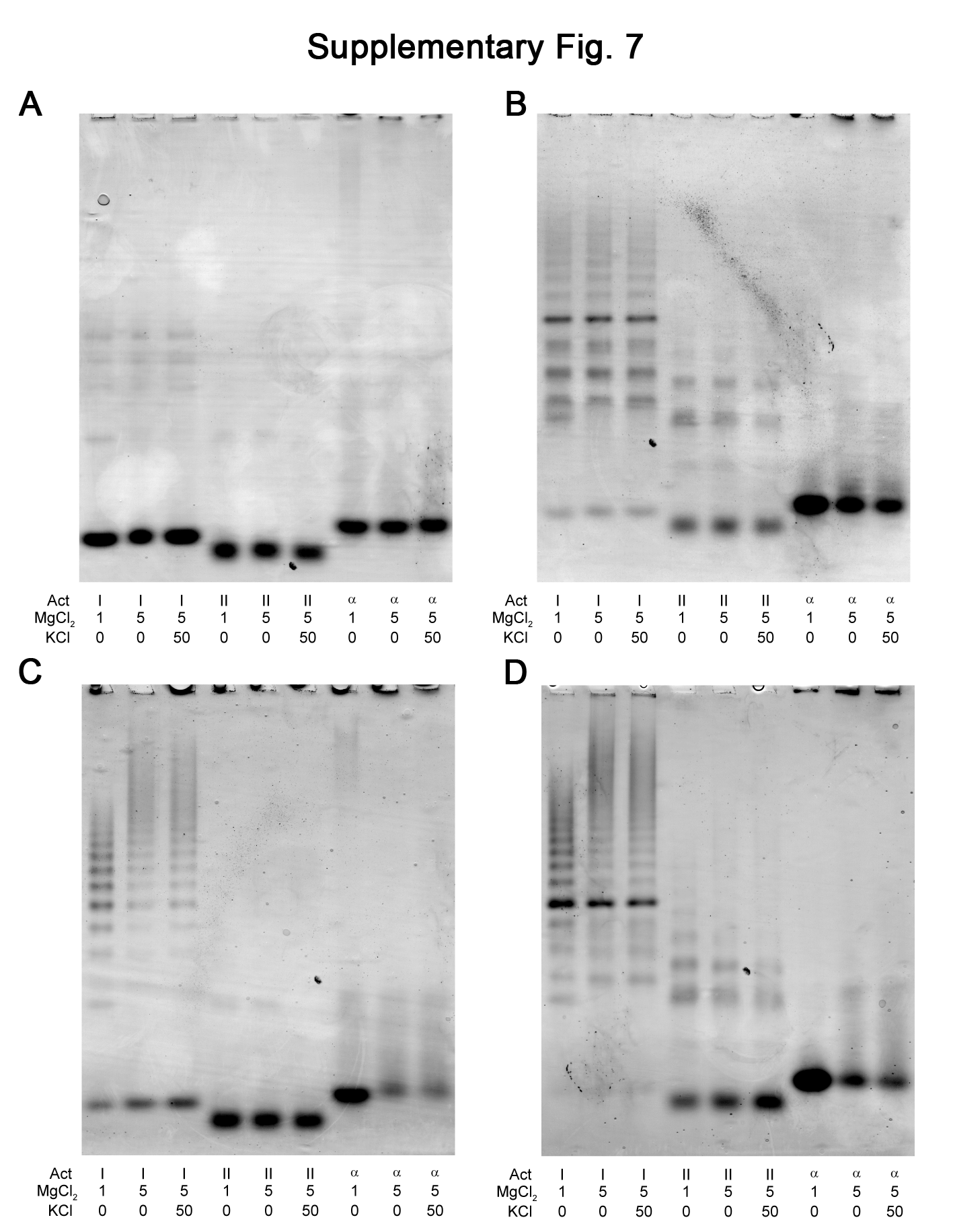

Supplement: Figure S7 — Behavior of Plasmodium actins and α-actin in native PAGE in the presence of magnesium and potassium. Native PAGE gels showing Plasmodium actins and α-actin in ATP (A, C) and ADP (B, D) forms 0 and 48 h after purification with either 1 mM MgCl2, 5 mM MgCl2 and 0.5 mM EGTA, or 5 mM MgCl2, 0.5 mM EGTA, and 50 mM KCl in the sample. (TIF) [file ppat.1004091.s007.tif]
